# Comprehensive LC-MS/MS analysis of nitrogen-related plant metabolites

**DOI:** 10.1093/jxb/erae129

**Published:** 2024-04-25

**Authors:** Sanja Ćavar Zeljković, Nuria De Diego, Lukáš Drašar, Jaroslav Nisler, Libor Havlíček, Lukáš Spíchal, Petr Tarkowski

**Affiliations:** Czech Advanced Technology and Research Institute, Palacky University, Šlechtitelů 27, 78371 Olomouc, Czech Republic; Centre of the Region Haná for Biotechnological and Agricultural Research, Department of Genetic Resources for Vegetables, Medicinal and Special Plants, Crop Research Institute, Šlechtitelů 29, 78371 Olomouc, Czech Republic; Czech Advanced Technology and Research Institute, Palacky University, Šlechtitelů 27, 78371 Olomouc, Czech Republic; Czech Advanced Technology and Research Institute, Palacky University, Šlechtitelů 27, 78371 Olomouc, Czech Republic; Isotope Laboratory, Institute of Experimental Botany, The Czech Academy of Sciences, Vídeňská 1083, CZ-14220, Prague, Czech Republic; Czech Advanced Technology and Research Institute, Palacky University, Šlechtitelů 27, 78371 Olomouc, Czech Republic; Isotope Laboratory, Institute of Experimental Botany, The Czech Academy of Sciences, Vídeňská 1083, CZ-14220, Prague, Czech Republic; Czech Advanced Technology and Research Institute, Palacky University, Šlechtitelů 27, 78371 Olomouc, Czech Republic; Czech Advanced Technology and Research Institute, Palacky University, Šlechtitelů 27, 78371 Olomouc, Czech Republic; Centre of the Region Haná for Biotechnological and Agricultural Research, Department of Genetic Resources for Vegetables, Medicinal and Special Plants, Crop Research Institute, Šlechtitelů 29, 78371 Olomouc, Czech Republic; University of South Bohemia in České Budějovice, Czech Republic

**Keywords:** Acetylated amino acids, acetylated biogenic amines, amino acids, biogenic amines, LC-MS/MS, methylated amino acids, plant metabolism

## Abstract

We have developed and validated a novel LC-MS/MS method for simultaneously analyzing amino acids, biogenic amines, and their acetylated and methylated derivatives in plants. This method involves a one-step extraction of 2–5 mg of lyophilized plant material followed by fractionation of different biogenic amine forms, and exploits an efficient combination of hydrophilic interaction liquid chromatography (HILIC), reversed phase (RP) chromatography with pre-column derivatization, and tandem mass spectrometry (MS). This approach enables high-throughput processing of plant samples, significantly reducing the time needed for analysis and its cost. We also present a new synthetic route for deuterium-labeled polyamines. The LC-MS/MS method was rigorously validated by quantifying levels of nitrogen-related metabolites in seedlings of seven plant species, including Arabidopsis, maize, and barley, all of which are commonly used model organisms in plant science research. Our results revealed substantial variations in the abundance of these metabolites between species, developmental stages, and growth conditions, particularly for the acetylated and methylated derivatives and the various polyamine fractions. However, the biological relevance of these plant metabolites is currently unclear. Overall, this work contributes significantly to plant science by providing a powerful analytical tool and setting the stage for future investigations into the functions of these nitrogen-related metabolites in plants.

## Teaser

A new high-throughput LC-MS/MS technique for analysis of amino acids and biogenic amines and their acetylated and methylated derivatives revealed substantial variations among plant species, developmental stages, and growth conditions.

## Introduction

Amino acids are small organic primary metabolites that play essential roles in the fixation of nitrogen and its inter- and intracellular transport in plants (Dellero, 2020). They accumulate during many biological processes, including plant growth and development, synthesis of peptides and proteins, and plant stress responses induced during antioxidative or plant defense responses (Podlešáková *et al.*, 2019). They also serve as precursors for other secondary metabolites such as polyamines, or biogenic amines, which are mainly polymer-like carbon chain structures containing multiple amino groups. The possible signaling roles of amino acids and polyamines have been discussed extensively in the recent scientific literature (Podlešáková *et al.*, 2019; Seifi and Shelp, 2019; Caspi *et al.*, 2023). Additionally, the potential roles of some amino acids and acetylated or methylated polyamines in plant biology have drawn interest in recent years, leading to suggestions that they may have functions similar to those seen in mammals (Bělíček *et al.*, 2023). However, few studies have investigated their biological activities in plants (Adio *et al.*, 2011; Jammes *et al.*, 2014; Abdelhakim *et al.*, 2022).

The simultaneous analysis of these nitrogen-containing plant metabolites is complex due to their structural diversity, high polarity, and lack of specific chromophores. Amino acids and their derivatives contain primary or secondary amino groups as well as carboxyl groups, making them amphoteric compounds (Violi *et al.*, 2020). Because of their importance and ubiquity, they have been analyzed in a wide variety of biological matrices (Thiele *et al.*, 2008; Wen *et al.*, 2019; Su *et al.*, 2021; Langner *et al.*, 2022), foods (Zhou *et al.*, 2013; Dong and Xiao, 2017), and environmental samples (Gao *et al.*, 2016). They are typically identified in their native form or via pre-column derivatization, which enables their detection using spectrophotometric methods such as UV/Vis spectroscopy or fluorescence. However, mass spectrometric (MS) techniques are preferred because they offer significantly greater sensitivity and selectivity in addition to providing structural information that facilitates identification of analytes.

The high polarity of these compounds means that they are either not retained or poorly separated on reversed phase (RP) chromatographic columns. Therefore, they are often derivatized before separation. Derivatization increases analysis times but has the benefit of increasing sensitivity. Common derivatization agents for amino acids are Fmoc (9-fluorenylmethyloxycarbonyl chloride), AQC (6-aminoquinolyl-*N*-hydroxysuccinimidyl carbamate), and PFC (propyl chloroformate) (Violi *et al.*, 2020), while biogenic amines are commonly derivatized with dansyl chloride, fluorescamine, *o*-phthaldialdehyde (OPA), or benzoyl, dansyl, or tosyl chlorides (Dai *et al.*, 2014). Another option is to use a mobile phase containing ion-pairing agents that form complexes with the analytes and increase their retention on RP columns. Pentafluorooctanoic acid or diisopropylethylamine have been used as ion-pairing agents for amino acids (Violi *et al.*, 2020), while pentafluoropropionic (PFA), heptafluoroheptanoic (HFBA), and perfluoroheptanoic (PFPA) acids have been used for biogenic amines (Häkkinen, 2011; Yu *et al.*, 2015). However, ion-pairing agents reduce detection limits and cause ion suppression in the mass spectrometer source.

Hydrophilic interaction liquid chromatography (HILIC) is a separation technique that can be combined with MS detection. It uses a polar stationary phase with a mobile phase consisting of mainly organic solvent and is suitable for separating polar compounds that are not retained on RP columns. As summarized by Ruta *et al.* (2010), the main advantages of HILIC are good retention of polar compounds and improved sensitivity due to the high content of organic solvent in the mobile phase, which weakens matrix effects and improves spraying conditions in the mass spectrometer while also reducing back pressure. A literature review indicates that HILIC is the most frequently used chromatographic technique for separating underivatized (i.e. native) amino acids (Guo *et al.*, 2013; Zhou *et al.*, 2013; Gao *et al.*, 2016; Prinsen *et al.*, 2016; Tsochatzis *et al.*, 2017; Thiele *et al.*, 2008; Wen *et al.*, 2019; Violi *et al.*, 2020) and has also recently been used to separate biogenic amines (Su *et al.*, 2021; Langner *et al.*, 2022). However, there are very few reports on the quantification of underivatized amino acids in plant tissue (Guo *et al.*, 2013; Zhou *et al.*, 2013; Thiele *et al.*, 2008) and the methods that have been used for this purpose have significant drawbacks, including requiring large amounts of plant material, long analysis times, high carryover, and narrow linear ranges.

Here, we report the development and validation of a method for studying nitrogenous plant metabolites that combines HILIC and RP separation with MS detection, showing that it can successfully detect 74 nitrogen-related plant metabolites, including amino acids and biogenic amines, as well as their acetylated and methylated derivatives.

## Materials and methods

### Chemicals

The complete list of chemicals and reagents used in this study is given in Appendix S1.

### Synthesis of deuterium-labeled biogenic amines

All chemicals were of analytical grade and purchased from regular commercial sources. TLC analysis was conducted using silica plates (Merck 60F_254_) and the results were visualized by examining UV absorption after spraying the plate with a solution of ninhydrin (0.4%) in a mixture of *n*-butanol:acetic acid (100:3) and then heating. Merck 60 silica gel (0.040–0.063 mm) was used for column chromatography. Melting points were determined on a Kofler apparatus and are reported uncorrected. Mass spectra were acquired on a Waters Micromass mass spectrometer equipped with a ZMD detector, direct inlet, electrospray ionization (ESI), and a coin voltage (CV). 1H NMR spectra were recorded on a 700 MHz Bruker AVANCE III NMR spectrometer.

#### Synthesis of diaminopropane-1,1,2,2,3,3-d_6_ dihydrochloride (2, d_6_-Dap)

Malonitrile (**1**) (0.7 g, 10.6 mmol) was dissolved in 90 ml of ethan-[^2^H]ol (15 ml). Deuterium chloride (^2^HCl 18% in ^2^H_2_O) and 2.5 g of 10% Pd/C were added and the mixture was vigorously agitated under an initial deuterium gas pressure of 0.4 MPa for 16 h. The mixture was then filtered to remove the catalyst, and the essential part of the product was eluted from the catalyst using ethanol–water (1:1). The solvents were removed under reduced pressure to give an off-white solid. Recrystallization of the product from water–ethanol yielded 1.05 g (65%) of product **2.** mp: 248–250 °C, MS ESI: 81 [M+ H^+^], isotopic composition (by MS-ESI) 78% ^2^H_6_, ^2^H_5_.

#### Synthesis of N,N-diacetyldiaminopropane-1,1,2,2,3,3-d_6_ (3, d_6_-dAcDap)

NaHCO_3_ (400 mg, 4.7 mmol) was added to the solution of diaminopropane-1,1,2,2,3,3-d_6_ dihydrochloride (98 mg, 0.64 mmol) in ethanol (4 ml). The mixture was stirred for 5 min, and acetic anhydride (0.43 ml, 3.89 mmol) was added dropwise for 60 min. The reaction mixture was stirred overnight at room temperature and monitored by TLC, eluting with 5% MeOH in CHCl_3_. A solution of product **3** was obtained by centrifuging the suspension. Column chromatography on SiO_2_ (5–20% EtOH in CHCl_3_) and crystallization from EtOAc gave 58 mg of pure product **3** (56%). mp: 94–97 °C, MS ESI: 165 [M+ H^+^], 187 [M+ Na^+^], 329 [2M+ H^+^], isotopic composition (determined by MS ESI): ^2^H_5_ 19%, ^2^H_6_ 81%. 1H NMR (700 MHz, CDCl_3_) δ ppm 1.79–2.13 (m, 1 H).

#### *Synthesis of* N*-acetyldiaminopropane-,1,2,2,3,3-d*_*6*_*hydrochloride (4, d*_*6*_*-AcDap)*

(^2^H_6_)Propane-1,3-diamine hydrochloride **2** (1104 mg, 9.47 mmol) was suspended in methanol (10 ml). The suspension was neutralized to pH 9 with NaOH (908 mg, 22.72 mmol). Dry acetic acid (10 ml) was added, the mixture was heated to 55 °C, and acetic anhydride (0.81 ml, 8.57 mmol) was added dropwise with stirring for 1 h. The mixture was then evaporated to dryness and the residue was dissolved in water and adjusted to pH <1 with 10 N HCl (2 ml), after which the mixture was again evaporated to dryness. The resulting solid was extracted with hot 2-propanol, and the insoluble residue was discarded. The filtrate was evaporated to dryness. Monoacetylated diamine **4** was obtained (613 mg, 45%) as pale-yellow crystals. MS ESI: 123 [M+ H^+^], 1H NMR (700 MHz, D_2_O) δ ppm 1.86 (s, 3 H) 1.86–1.86 (m, 1 H).

#### *Synthesis of* N*-{3-[(4-nitrobenzene-1-sulfonyl)amino](*^*2*^*H*_*6*_*)propyl}acetamide (5*)

Na_2_CO_3_ (223 mg, 2.1 mmol) and 4-nitrobenzene-1-sulfonyl chloride (433 mg, 2 mmol) were added to a solution of *N*-[3-amino(^2^H_6_)propyl]-acetamide hydrochloride **4** (182 mg, 1.5 mmol) in dichloromethane and the mixture was stirred overnight at room temperature. Water (10 ml) was then added and the suspension was extracted with ethyl acetate (3 × 15 ml). The combined organic layers were collected and evaporated, yielding a residue that was diluted in water (70 ml) at 95 °C, filtered, and slowly cooled to room temperature. The crystals that formed were collected by filtration. Sulfonamide **5** (335 mg, 73%) was obtained as white crystals. MS ESI: 308 [M+ H^+^], 330 [M+ Na^+^]. 1H NMR (700 MHz, dimethyl sulfoxide-d_6_) δ ppm 1.76 (s, 3 H) 8.04 (d, J=8.37 Hz, 2 H) 8.43 (d, J=8.64 Hz, 2 H).

#### *Synthesis of* tert*-butyl(4-{[3-acetamido(*^*2*^*H*_*6*_*)propyl](4-nitrobenzene-1-sulfonyl)amino}butyl)carbamate (8*)

Compound **5** (227 mg, 0.739 mmol) was dissolved in dry tetrahydroxyfuran (15 ml). Compound **6** (384 mg, 1.11 mmol), triphenylphosphane (388 mg, 1.48 mmol), and diisopropyl azodicarboxylate (299 mg, 1.48 mmol) were then added and the reaction mixture was stirred overnight with monitoring by TLC (CHCl_3_/MeOH 1:5). The solvent was then evaporated and the residue was diluted in dichloromethane (20 ml), washed with water (20 ml) and brine (20 ml), dried over MgSO_4_, filtered, and evaporated under reduced pressure. The crude product was purified by column chromatography on silica gel (CHCl_3_/MeOH 0–8% MeOH), giving compound **6** as a colorless oil (341 mg, 98%). MS ESI: 636 [M+ H^+^].

#### *Synthesis of* tert*-butyl (4-{[3-acetamido(*^*2*^*H*_*6*_*)propyl](4-nitrobenzene-1-sulfonyl)amino}butyl){2-[(*tert*-butoxycarbonyl)amino]ethyl}carbamate (9*)

Compound **5** (394 mg, 1.28 mmol) was dissolved in dry tetrahydroxyfuran (15 ml). Compound **7** [533 mg, 1.54 mmol, prepared by the method of Lebreton *et al.* (1999)], triphenylphosphane (404 mg, 1.54 mmol), and diisopropyl azodicarboxylate (311 mg, 1.54 mmol) were then added and the reaction mixture was stirred overnight with monitoring by TLC (CHCl_3_/MeOH 1:5). The solvent was then evaporated and the residue was diluted in dichloromethane (20 ml), washed with water (20 ml) and brine (20 ml), dried over MgSO_4_, filtered, and evaporated under reduced pressure. The crude product was purified by column chromatography on silica gel (CHCl_3_/MeOH 0–8% MeOH), giving compound **6** as a colorless oil (775 mg, 95%). MS ESI: 636 [M+ H^+^].

#### *Synthesis of* tert*-butyl (4-{[3-acetamido(*^*2*^*H*_*6*_*)propyl]amino}butyl)carbamate (10*)

K_2_CO_3_ (361 mg, 3.62 mmol) and thiophenol (203 μl, 2.18 mmol) were added to a solution of compound **8** (335 mg, 0.70 mmol) in dry dimethylformamide (DMF; 3 ml) at 0 °C. The reaction mixture was stirred overnight and monitored by TLC (CHCl_3_/MeOH 1:5). The solvent was evaporated and the residue was poured into H_2_O (20 ml) and extracted with EtOAc (2 × 15 ml). The organic extracts were evaporated and diluted in EtOH/H_2_O (20 ml, 4:1). Ion exchanger (2 ml) in H^+^ form was added to the solution and the mixture was stirred for 1 h. The solvent was filtered off and the ion exchanger was washed with EtOH/H_2_O (60 ml, 4:1). The ion exchanger was then eluted with EtOH/H_2_O/25% aq NH_4_OH (60 ml, 30:15:1) and the solvent was evaporated to yield product **10** as a white foam (120 mg, 58%). MS ESI: 294 [M+ H^+^]; 292 [M– H^+^].

#### *Synthesis of* tert*-butyl (4-{[3-acetamido(*^*2*^*H*_*6*_*)propyl]amino}butyl){2-[(tert-butoxycarbonyl)amino]ethyl}carbamate (11*)

K_2_CO_3_ (804 mg, 5.83 mmol) and thiophenol (454 μl, 4.85 mmol) were added to a solution of compound **9** (775 mg, 1.62 mmol) in dry DMF (5.00 ml) at 0 °C. The reaction mixture was stirred overnight and monitored by TLC (CHCl_3_/MeOH 1:5). The solvent was evaporated and the residue was poured into H_2_O (20 ml) and extracted with EtOAc (2 × 20 ml). The organic extracts were evaporated and diluted in ethanol/water (30 ml, 4:1). Ion exchanger (3 ml) in H^+^ form was added to the solution and the mixture was stirred for 1 h. The solvent was filtered off and the ion exchanger was washed with ethanol/water (60 ml, 4:1) before being eluted with EtOH/H_2_O/25% aq NH_4_OH (60 ml, 30:15:1). Finally, the solvent was evaporated and the crude product (277.8 mg) was purified by column chromatography on silica gel (CHCl_3_:MeOH 5:1) to yield compound **11** as a white foam (147 mg, 31%). MS ESI: 451 [M+ H^+^], 1H NMR (700 MHz, CHLOROFORM-d) δ ppm 1.45 (s, 9 H) 1.48 (s, 9 H) 1.62–1.74 (m, 4 H) 1.79–1.88 (m, 2 H) 2.05 (s, 3 H) 3.07–3.15 (m, 2 H) 3.16–3.33 (m, 6 H).

#### *Synthesis of* N*1-acetylspermidine-2,2,3,3,4,4-d*_*6*_*dihydrochloride (12, d*_*6*_*-NAcSpd)*

Compound **10** (113 mg, 0.12 mmol) was dissolved in 4 M HCl in dioxane (10 ml) and stirred at 25 °C overnight. The solvent was then evaporated *in vacuo* and the residue was evaporated with absolute EtOH (3 × 20 ml) and lyophilized in dioxane to afford *N*1-acetylspermidine-2,2,3,3,4,4-d_6_ dihydrochloride **12** (80 mg, 78%). MS ESI: 195 [M+ H^+^], 1H NMR (700 MHz, D_2_O) δ ppm 1.57–1.71 (m, 4 H) 1.87 (s, 3 H) 2.88–2.92 (m, 2 H) 2.93–2.97 (m, 2 H).

#### *Synthesis of* N*1-acetylspermine-2,2,3,3,4,4-d*_*6*_*trihydrochloride (13, d*_*6*_*-NAcSpm)*

Compound **11** (52 mg, 0.12 mmol) was dissolved in 4 M HCl in dioxane (10 ml) and stirred at 25 °C overnight. The solvent was then evaporated *in vacuo* and the residue was evaporated with absolute EtOH (3 × 20 ml) and lyophilized in dioxane to afford 1,1,2,2,3,3-^2^H_6_-spermine tetrahydrochloride **13** (35 mg, 84%). MS ESI: 251 [M+ H^+^], 1H NMR (700 MHz, D_2_O) δ ppm 1.75 (br s, 4 H) 1.98 (s, 3 H) 2.04–2.10 (m, 2 H) 3.03–3.11 (m, 6 H) 3.12–3.17 (m, 2 H).

#### *Synthesis of* N*-(3-aminopropyl)acetamide (AcDap)*

The title compound was prepared and purified using a protocol analogous to that described above for **4** (d_2_-AcDap). MS ESI: 117 [M+ H^+^], 1H NMR (500 MHz, dimethyl sulfoxide-d_6_) δ ppm 1.56–1.71 (m, 2 H) 1.77 (s, 3 H) 2.65–2.78 (m, 2 H) 2.98–3.11 (m, 2 H) 7.97–8.18 (m, 3 H).

### Analyte solutions

Stock solutions (100 mM) of all analytes and internal standards were prepared by dissolving the appropriate mass of the analyte in 0.1 M HCl. Asn (asparagine), Glu (glutamic acid), Gln (glutamine), tSpm (thermospermine), Spm (spermine), Spd (spermidine), hSpd (*homo*-spermidine), Cad (cadaverine), Agm (agmatine), and Put (putrescine) were dissolved in milliQ water (Millipore, Germany). These stock solutions were kept at –20 °C. Amino acid solutions were stable for 2 months, while biogenic amines were stable for 2 weeks of continuous use. The working solutions (0.005–250 μM) were prepared in MeOH or the relevant mobile phases.

### Ultra-high performance–tandem mass spectrometry analyses

Analyses were performed on a Nexera X2 UHPLC instrument (Shimadzu Handels GmbH) coupled to an MS-8050 mass spectrometer (Shimadzu Handels GmbH). Chromatographic separation was performed using Acquity UPLC BEH C18 (50 × 2.1 mm; 1.7 µm particle size) or Acquity UPLC BEH AMIDE (150 × 2.1 mm; 1.7 µm particle size) analytical columns with suitable pre-columns. Columns were held at 40 °C and the flow rate was 0.4 ml min^–1^ for the BEH C18 column and 0.35 ml min^–1^ for the BEH AMIDE column. The injection volume was 2 μl for the 50 mm column and 10 μl for the 150 mm column.

The mobile phase for the RP BEH C18 column was a mixture of aqueous solutions of 15 mM formic acid (FA), pH 3.0 (adjusted with NH_4_OH) (component A) and MeOH (component B), while the mobile phase for both HILIC and the BEH AMIDE column was a mixture of 15 mM FA, pH 3.0 (adjusted with NH_4_OH) (component A) and 0.1% FA in ACN (acetonitrile) (component B). Gradient elution with the BEH C18 column was performed under the following conditions: 0 min 30%, 0.5 min 57% B, 3 min 57% B, 3.5 min 90% B, isocratic 90% B for 0.5 min, 4.1 min 30% B, and equilibration for the next 2.4 min. Gradient elution with the BEH AMIDE column was done using the following regime: 0–2 min 95% B, 10 min 75% B, 14 min 55% B, 17.5 min 20% B, 17.6 min 10% B, isocratic 10% B for 1.9 min, 20.5 min 95% B, and equilibration for the next 9.5 min. A diverter valve was used to automatically divert salt and buffer solutions from the mobile phase to waste at the beginning of the run and highly hydrophobic compounds not relevant to this work at the end of each run.

Mass spectra for all analytes except allantoin (All) were obtained via ESI in positive mode with the following operating parameters: capillary voltage –3 kV, interface voltage 4 kV, interface temperature 300 °C, heating and drying gas ﬂow 10 l min^–1^, nebulizing gas ﬂow 3 l min^–1^. The MRM (multiple reaction monitoring) transitions of underivatized compounds are summarized in Supplementary Table S1, and those for benzoylated biogenic amines are given in Supplementary Table S2. Analytes were identified by comparing their retention times, MRM transitions, and relative intensities with those of authentic standards, while the isotope dilution method was used for quantification (Rittenberg and Foster, 1940).

### Biological material

**Fig. 1.**
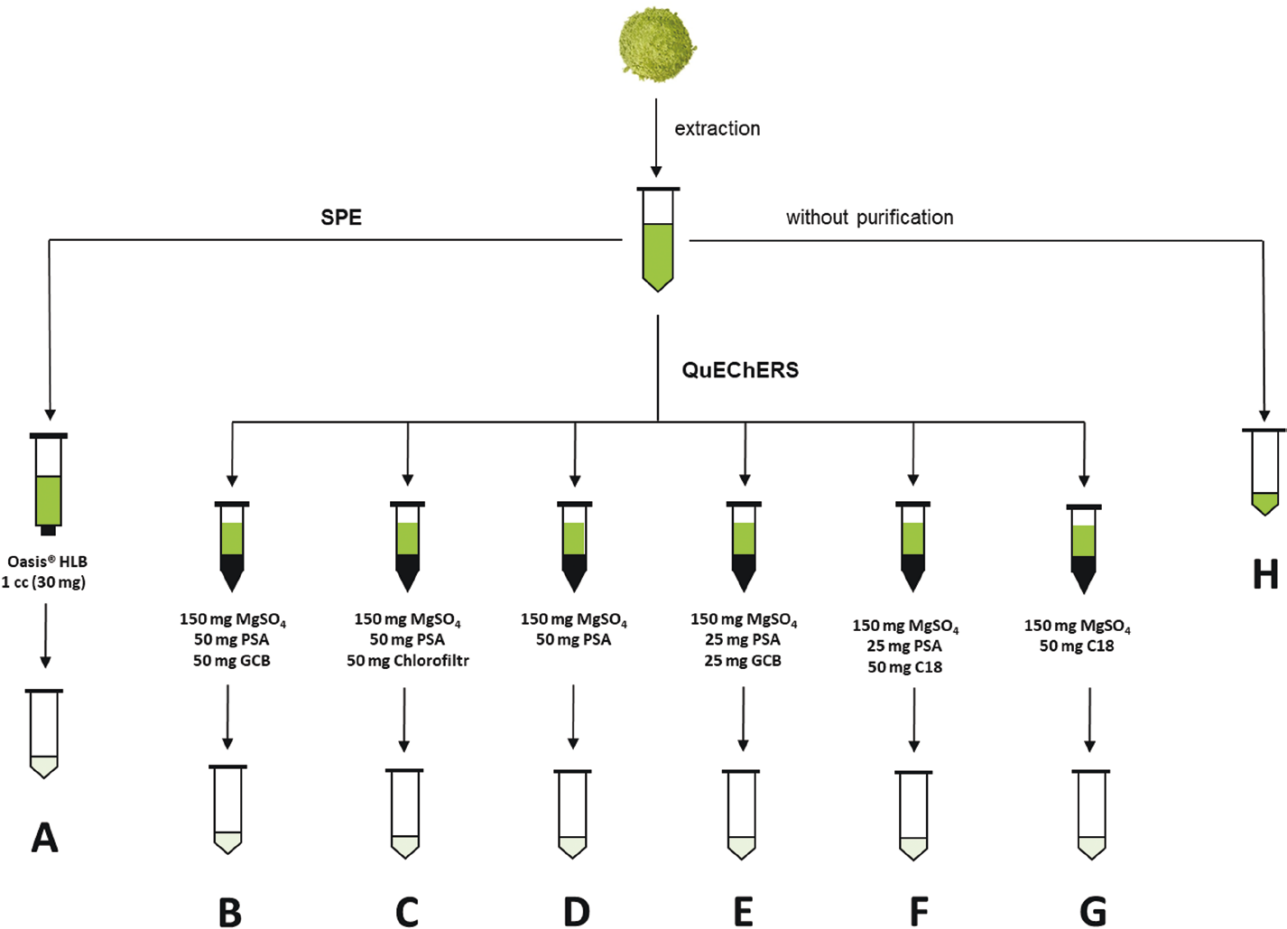
Development of the extraction and purification protocol. (A) Oasis HLB 1 cc (30 mg) cartridges; (B) roQ QuEChERS dSPE 150 mg of MgSO_4_/50 mg of PSA/50 mg GCB; (C) UCT QuEChERS 150 mg of MgSO_4_/50 mg of PSA/50 mg of Chlorofiltr; (D) DisQuE 150 mg of MgSO_4_/50 mg of PSA; (E) DisQuE 150 mg of MgSO_4_/25 of mg PSA/25 mg of GCB; (F) DisQuE 150 mg of MgSO_4_/25 mg of PSA/50 mg of C18; (G) DisQuE 150 mg of MgSO_4_/50 mg of C18; (H) without purification.

Seeds of the following plants were used in this work: *Arabidopsis thaliana* (L.) Heynh. accession Columbia-0, as the most used model plant, radish (*Raphanus sativus* L.), maize (*Zea mays* L.), barley (*Hordeum vulgare* L. cv. ‘Francin’), wheat (*Triticum aestivum* L. v. ‘Turandot’), tomato (*Solanum lycopersicum* L. cv. ‘Daniela’), and tobacco (*Nicotiana tabacum* L.). For Arabidopsis, seeds were sown in square plates supplemented with 1/2 Murashige and Skoog (MS) medium until they were 3 days old, as described by De Diego *et al.* (2017). The plants were then transferred to plastic pots (70 mm×70 mm×65 mm, Poppelmann, TEKU, Germany) containing 130 g of freshly sieved soil (Substrate 2; Klasmann-Deilmann GmbH, Geeste, Germany) and placed in a growing chamber under controlled conditions with a light intensity of 120 μmol photons s^–1^ m^–2^ and a 16/8 h light regime at a temperature of 22/20 °C and 60% relative humidity. The Arabidopsis seedlings were grown under two water regimes: control and stressed plants were watered to 80% and 50% of the full soil water capacity, respectively, for 4 weeks. Rosettes from 20 plants per variant were then collected for protocol development (Fig. 1). The remaining plants were transferred to pots filled with the same amount of substrate and grown under the same conditions. The aerial parts of radish, maize, barley, and wheat seedlings were collected after 8 d, while those of tomato plants were collected after 15 d. The collected tomato aerial parts were separated into two groups: those with only cotyledons and those with a visible true leaf. Tobacco seedlings were collected after 25 d. Collected samples were immediately frozen in liquid nitrogen, stored at –80 °C, lyophilized (Martin Christ Beta 1-8 LDplus, Germany) to complete dryness, and homogenized in a mixer mill (Retch, Germany). All homogenized samples were stored at –20 °C until extraction.

### Optimization of the extraction and purification protocol

Homogenized Arabidopsis plant tissue samples (5 mg) were extracted with 1.8 ml of (a) 96% EtOH, (b) 5% FA in 96% EtOH, (c) 70% EtOH, (d) 5% FA in 70% EtOH, (e) 50% EtOH, (f) 5% FA in 50% EtOH, and (g) 5% trichloroacetic acid, following a previously reported protocol for extracting the metabolites of interest (Taibi *et al.*, 2000). Three technical replicate samples were extracted with each solvent. After sonication for 15 min, the extracts were centrifuged for 5 min at 25 °C and 14 500 *g*, and the supernatant was collected in a new vial.

To optimize the purification protocol, 150 μl of supernatant was mixed with 1350 μl of ACN, and the solution was purified using solid-phase extraction (SPE) or QuEChERS (quick, easy, cheap, effective, rugged, and safe) cartridges as shown in Fig. 1A–G. Additional non-purified extracts were kept as negative controls (Fig. 1H).

For the first purification method, SPE cartridges (Fig. 1A) were activated with 1 ml of MeOH and 1 ml of milliQ water, then equilibrated with 1 ml of ACN. Next, a sample containing 150 μl of plant extract and 1350 μl of ACN was loaded onto the cartridge, which was then washed with 1 ml of ACN. The loading and washing fractions were collected and combined, evaporated under vacuum to dryness at 40 °C, reconstituted in 25 μl of the mobile phase, and analyzed by UHPLC-MS/MS.

For the second purification method, 150 μl of plant extract was loaded onto QuEChERS cartridges with different compositions as specified in Fig. 1B–G. The loading fraction was then vortexed for 10 s and centrifuged for 5 min at 25 °C at 14 500 *g*, after which the supernatant was collected in a new vial. After evaporation under vacuum to dryness at 40 °C, the extract was reconstituted in 25 μl of the mobile phase and analyzed by UHPLC-MS/MS.

The extraction and purification protocol that achieved the best performance for all of the compounds of interest was applied to tissue samples from seven plant species, including Arabidopsis. Tissue samples (2–5 mg) comprising lyophilized material from the aerial parts of each plant species were extracted with 1 ml of 50% EtOH; three biological replicates were used for each individual plant included in the analysis. After sonication for 15 min, each extract was centrifuged for 5 min at 25 °C at 14 500 *g*, then three 250 μl samples of the supernatant were placed in different vials. Sample I was used for analysis of free amino acids and methylated and acetylated metabolites, while samples II and III were used for analysis of free and conjugated biogenic amines.

Sample I was evaporated to dryness and redissolved in 50 μl of mobile phase for HILIC separation on the BEH AMIDE column to quantify free amino acids and biogenic amines and their acetylated and methylated derivatives, as well as selected free nitrogen-containing compounds. Sample II was subjected to the derivatization protocol of Taibi *et al.* (2000) with slight modifications as described below. After evaporation to dryness, the sample was dissolved in a mobile phase for RP separation on the BEH C18 column to quantify free biogenic amines. Sample III was subjected to acid hydrolysis (100 μl of 12 M HCl) for 16 h at 40 °C to liberate conjugated biogenic amines (Piqueras *et al.*, 2002). After derivatization, as described below, the sample was prepared for analysis on the RP column in the same way as sample II. As a result, sample III contained both free and conjugated biogenic amines whereas sample II contained only free biogenic amines. The concentrations of conjugated amines were thus determined by subtracting the values obtained for sample II from those for sample III.

The pellet that remained after extraction was hydrolyzed by treatment with 200 μl of 6 M NaOH under sonication for 15 min, followed by heating at 40 °C in 200 μl of 12 M HCl to release macromolecule-bound biogenic amines. After derivatization, the resulting sample IV was prepared for analysis on the RP column.

### Derivatization of biogenic amines

To enable accurate analysis of biogenic amines, derivatization was performed by adding 100 µl of 6 M NaOH (300 µl for hydrolyzed samples) to samples II, III, and IV along with 50 μl of 10 nM d_6_-Dap (diaminopropane-1,1,2,2,3,3-d_6_), followed by 2.5 µl of benzoyl chloride (in MeOH, 50:50, v:v). After vortexing for 5 s, the reaction mixtures were stirred for 40 min at 25 °C, then 500 µl of saturated NaCl was added and the benzoylated biogenic amines were extracted with 2 × 500 µl of diethyl ether. The solvent was evaporated under vacuum at 40 °C and the dry samples were dissolved in 250 μl of the mobile phase, sonicated for 10 min, and centrifuged for an additional 5 min at 14 500 *g*. Finally, the supernatant was transferred to a vial and analyzed. Standards of biogenic amines at concentrations of 1 μM (see Supplementary Table S2) were also derivatized using this protocol.

### Method validation

The method was validated for selectivity, linearity, limit of detection (LOD) and limit of quantification (LOQ), accuracy and precision (interday and intraday), matrix effects, process efficiency, carryover, and stability in accordance with the ICH M10 guideline on bioanalytical method validation and study sample analysis (ICH, 2022).

Selectivity and specificity were evaluated based on the chromatographic and MS characteristics of each target analyte and internal standards (Supplementary Tables S1, S2). Calibration curves were constructed by plotting the peak areas of the reference standards of target analytes against their concentrations using the isotope dilution analysis method (Jonckheere *et al.*, 1983). Each calibration curve was based on data for 10 concentration levels, each represented by triplicate samples. The LOD and LOQ for each analyte were defined as the concentrations giving a signal-to-noise ratio (S/N) of ~3 and 10, respectively. Intra- and inter-day variations were measured to evaluate the precision of the developed method. For the intra-day test, plant material enriched with three different analyte concentrations was analyzed using three replicates on the same day, while inter-day variability was evaluated using the same samples (analyzed in triplicate) over three consecutive days. These samples were also used to evaluate stability. Variation was quantified using the relative standard deviation (RSD). Matrix effects and process efficiency were evaluated by pre-extraction and post-extraction addition of internal standards (Matuzewski *et al.*, 2003). Carryover was tested by analyzing blank solvent immediately after analyzing a quantification sample at the highest concentration used when generating calibration curves.

### Statistical analysis

Data were analyzed using multivariate statistical analyses performed in RStudio (Version 1.1.463 – © 2009–2018 RStudio, Inc.) and Orange v. 3.31.1. Data were normalized using natural logarithm before analysis. Principal component analyses (PCAs) were carried out using the packages factoextra, factoMineR, and ggplot2.

## Results and discussion

### Synthesis of deuterium-labeled biogenic amines

Deuterated standards were prepared by reducing propanedinitrile **1** with deuterium under palladium catalysis. The resulting diaminopropane-1,1,2,2,3,3-d_6_ (**2**, d_6_-Dap) was acetylated with acetic anhydride in acetic acid to afford *N*,*N*-diacetyldiaminopropane-1,1,2,2,3,3-d_6_ (**3**, d_6_-dAcDap). *N*-Acetyldiaminopropane-,1,2,2,3,3-d_6_ (**4**, d_6_-AcDap) was prepared from the diaminopropane-1,1,2,2,3,3-d_6_ (**2**) by selective monoacetylation with acetic anhydride in acetic acid according to the method of Tabor *et al.* (1964). This amine was subsequently converted to sulfonamide **5** by reaction with paranitrobenzenesulfonylchloride.

Other deuterated polyamine standards were prepared using the Fukuyama–Mitsunobu reaction (Fukuyama *et al.*, 1995) between sulfonamide **5** and alcohols **6** or **7**. Compound **6** is commercially available; intermediate **7** was prepared according to Lebreton *et al.* (1999).

**Fig. 2.**
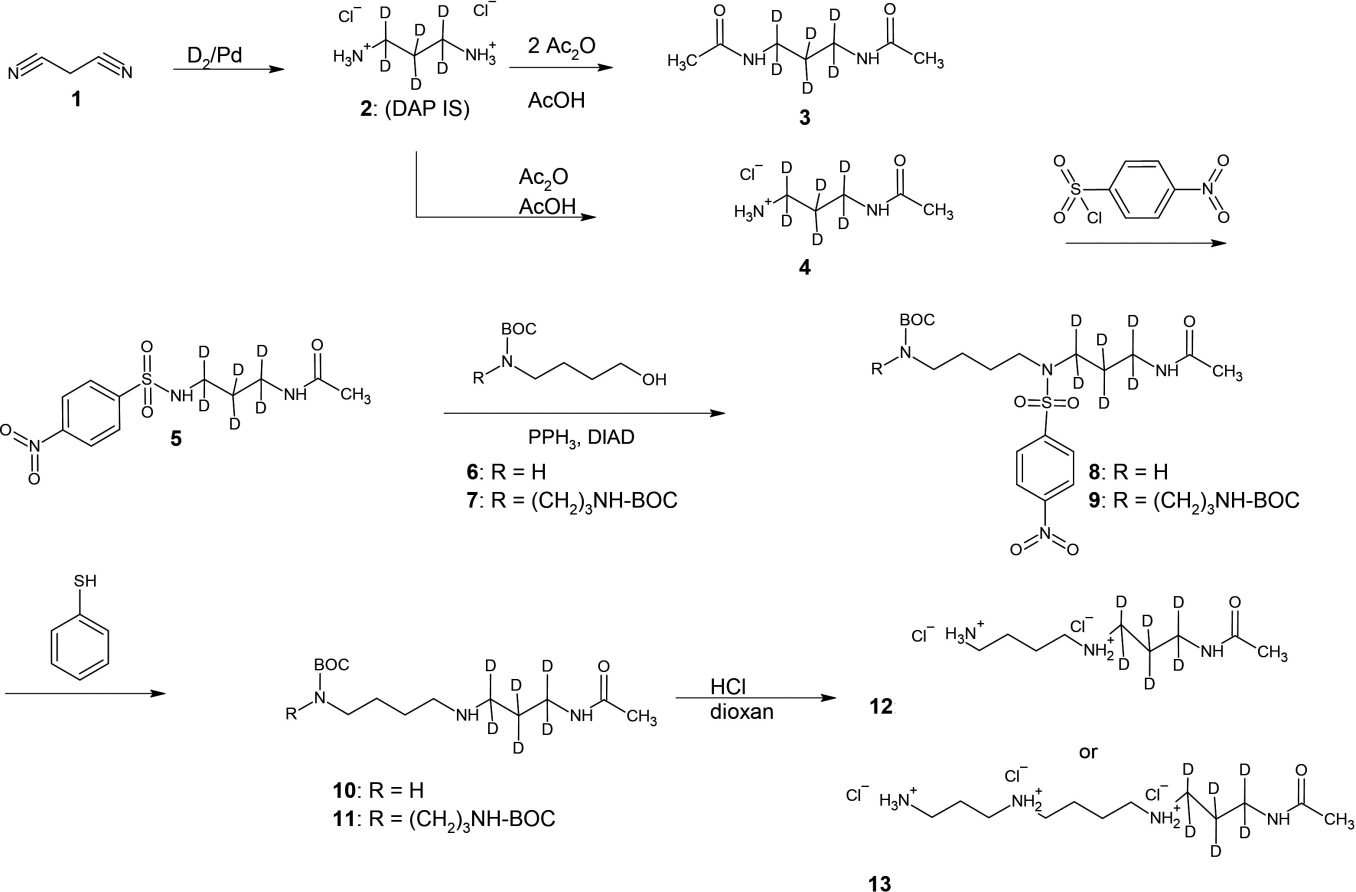
Synthesis of deuterium-labeled biogenic amines.

This efficient method using sulfonamide **5** enabled the preparation of key intermediates **8** and **9** in high yield and purity. The benefit of this method is that compounds **8** and **9** have strong UV chromophores and are thus readily analyzed by HPLC or TLC, their low polarity permits easy purification using SiO_2_ columns, and the nitrobenzenesulfonamide group can be selectively removed by stirring with thiophenol to obtain amines **10** and **11**. Another advantage of this strategy is that it permitted selective purification of amines **10** and **11** using an acidic ion exchange procedure. *N*1-acetylspermidine-2,2,3,3,4,4-d_6_ (**12**, d_6_-N1AcSpd) and *N*1-acetylspermine-2,2,3,3,4,4-d_6_ (**13**, d_6_-N1AcSpm) were obtained by deprotection of BOC group(s) using a 4 M solution of HCl in dioxane. All deuterated amines were prepared as stable hydrochloride salts (Fig. 2).

### MS/MS optimization

Because the amino group can easily form positive ions while the carboxyl group can form a stable negative ion, all compounds used in this study were first analyzed by direct full scan MS in both positive and negative ESI modes (Thiele *et al.*, 2008; Zhou *et al.*, 2013; Tašev *et al.*, 2017; Langner *et al.*, 2022). All analytes (10 μM) were introduced into the mass spectrometer using neutral (water:organic solvent, 50:50) and acidified mobile phases. The highest sensitivity was achieved using a mobile phase consisting of 15 mM FA (pH 3, adjusted with NH_4_OH), so this mixture was used in all subsequent experiments. Except for All, all analytes produced stronger responses in positive mode; this is consistent with the literature because Ohtake *et al.* (2004) also analyzed All in negative mode and free amino acids in positive mode. FA also suppressed the formation of molecular adducts with sodium and potassium, allowing analytes to be characterized based on the quasi-molecular [M+H]^+^ ions (or [M–H]^–^ for All). We next optimized several MS parameters including the choice of precursor and product ions, the collision energies, and the Q1 and Q3 pre-rod biases. Each compound was characterized by the two or three MRM transitions giving the strongest responses to ensure maximal sensitivity and selectivity. The results of the optimization experiments are summarized in Supplementary Table S1. Similar MRM optimization was performed for benzoylated derivatives of selected biogenic amines; their MRM transitions are summarized in Supplementary Table S2.

The most abundant product ion for most amino acids was [M+H-46]^+^, corresponding to the neutral loss of FA by rearrangement. Product ions corresponding to neutral loss of ammonia (i.e. [M+H-17]^+^) and both functional groups (i.e. [M+H-46-17]^+^) were also observed for these compounds. All these transitions are consistent with literature data (Thiele *et al.*, 2008; Guo *et al.*, 2013; Zhou *et al.*, 2013; Gao *et al.*, 2016). All of the acetylated amino acids formed stable pyrrolidone (*m/z* 84) or cyclic ammonium ions (*m/z* 70) resulting from functional group loss. Similar behavior was observed for Glu, Gln, and other essential amino acids such as Arg and Lys (Thiele *et al.*, 2008). MRM transitions of biogenic amines included characteristic product ions formed by neutral loss of ammonia, namely [M+H-17]^+^, as well as the [M+H-(CH_2_)_3_N_2_H_4_-NH_3_]^+^ fragment (*m/z* 112) that is specific for Spm (Dong and Xiao, 2017). Loss of the acetyloxy ion (*m/z* 59) was characteristic of most acetylated biogenic amines (Supplementary Table S1). The larger acetylated biogenic amines [AcSpm, AcNSpd (*N*-acetylnorspermidine), and dAcSpm (*N*1,*N*12-diacetylspermine)] also underwent C–N bond breakage to form the product ion *m/z* 100, in accordance with a recent report (Su *et al.*, 2021). Finally, the main MRM transition of benzoylated biogenic amines involved loss of the benzoyl cation (*m/z* 105) (Supplementary Table S2).

### Optimization of chromatographic conditions

HILIC has become popular in bioanalytical applications over the last two decades (Buszewski and Noga, 2012). In particular, HILIC is the most commonly used chromatographic method for analyzing free amino acids, which is done using stationary phases, including amides, silica, and materials with zwitterionic functional groups such as sulfobetaines (Violi *et al.*, 2020). The stationary phase of AQUITY UPLC BEH Amide columns contains trifunctional amide groups suitable for separating polar compounds such as amino acids and biogenic amines; columns of this type have therefore been used in previous studies on nitrogenous plant metabolites (Guo *et al.*, 2013; Zhou *et al.*, 2013; Gao *et al.*, 2016; Prinsen *et al.*, 2016; Tsochatzis *et al.*, 2017; Wen *et al.*, 2019; Su *et al.*, 2021). The works cited above generally examined ~20 compounds and used 100 mm long columns. However, in this work, we aimed to separate >70 metabolites, several of which have identical mass spectra. We, therefore, used a 150 mm long column.

The mobile phase for HILIC typically consists of a mixture of ACN and ammonium formate or ammonium acetate buffer at a concentration of 5–20 mM and a pH of ~3 (Langner *et al.*, 2022; Gao *et al.*, 2016; Guo *et al.*, 2013; Prinsen *et al.*, 2016; Tsochatzis *et al.*, 2017; Tašev *et al.*, 2017; Zhou *et al.*, 2013). Some authors have used significantly higher buffer concentrations of up to 50 mM (Thiele *et al.*, 2008), while others have used non-buffered mobile phases that were acidified with FA at concentrations of up to 0.2% (Wen *et al.*, 2019; Su *et al.*, 2021). High buffer concentrations can cause ion suppression and reduce the sensitivity of the analytical method. However, buffers are beneficial because the retention of ionizable compounds is very sensitive to the mobile phase pH. Based on the results of Gao *et al.* (2016) and Zhou *et al.* (2013), we tested FA buffer concentrations of 10, 15, and 20 mM (pH 3, adjusted with ammonium hydroxide) and found that the best overall sensitivity and reproducibility were obtained with a concentration of 15 mM (data not shown). This concentration was, therefore, used in all subsequent experiments. To minimize variation in the ESI efficiency during gradient elution (Liigand *et al.*, 2017), the ACN used as the organic component of the mobile phase was also acidified with 0.1% FA.

**Fig. 3.**
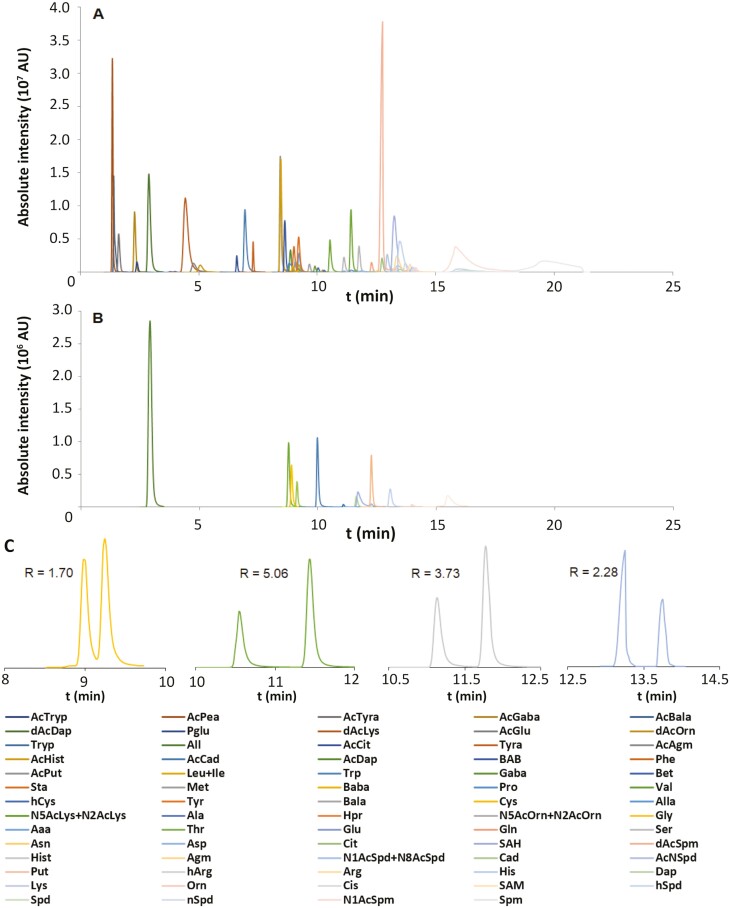
MS chromatograms. Separation of 73 nitrogen-containing compounds (A) and deuterium-labeled internal standards (B), including separation of isomers with identical MS/MS transitions (C), namely leucine and isoleucine, *N*6- and *N*2-acetyllysine, *N*5- and *N*2-acetylornithine, *N*1- and *N*8-acetylspermidine. All peaks presented here show transitions suitable for use as quantifiers. R denotes the chromatographic resolution.

By adjusting the chromatographic gradient, >70 compounds, including amino acids and their derivatives, biogenic amines and their derivatives, All, and allantoic acid (Alla) were successfully separated in 35 min, including the equilibration time (Fig. 3). The set of compounds included four pairs of isomers with identical MRM transitions, all of which were successfully separated with a chromatographic resolution of *R* >1.5 (Fig. 3).

Compounds with similar measured concentrations frequently had very different peak intensities that resulted from differences in their chemical structure and ionization in the mass spectrometer (Fig. 3A, B). In general, the peak intensities of the analyte groups decreased in the following order: acetylated biogenic amines>acetylated amino acids>amino acids>biogenic amines, in accordance with the LOD and LOQ values listed in the ‘Method validation’ section.

The use of appropriate internal standards (ISs) makes it possible to account for analyte loss during sample preparation and analysis. IS addition before sample extraction enables detection and quantitation of losses during sample preparation, while IS addition after sample preparation normalizes the analysis and makes it possible to detect changes in analyte behavior due to matrix effects as well as changes in instrument sensitivity. Although many ISs for amino acids and biogenic amines are commercially available, some published methods have only been validated using a small group of selected ISs (Thiele *et al.*, 2008; Gao *et al.*, 2016; Violi *et al.*, 2020; Su *et al.*, 2021). As shown in Fig. 3B, 12 deuterium-labeled ISs were used in this work: seven amino acids, one biogenic amine, and four acetylated biogenic amines. The chromatographic retention times of these ISs span the entire chromatogram, which is vital for detecting matrix effects and other factors that may influence the analysis as described above.

Some biogenic amines such as nSpd (norspermidine), Spd, and Spm had very high peak widths in excess of 2 min. Dong and Xiao (2017) reported similar issues when using the ACQUITY BEH HILIC column. While there are some reports describing the successful separation of selected biogenic amines using HILIC with a similar mobile phase composition (Su *et al.*, 2021; Langner *et al.* 2022), the elution gradients used in those works generally began with a mobile phase whose content of the aqueous component was at least 15%. Unfortunately, due to the low retention of some acetylated compounds [AcTryp (*N*-acetyltryptamine), AcPEA (*N*-phenethylacetamide), AcTyra (*N*-acetyltyramine), AcGABA (*N*-acetamidobutyric acid), AcBAla (*N*-acetyl-β-alanine), and dAcDap (*N*,*N*-diacetyldiaminopropane)], we were obliged to begin our gradient with an aqueous component content of 10%. An alternative approach was adopted by Häkkinen (2011) and Yu *et al.* (2015), who developed methods for separating underivatized biogenic amines using the ion-pairing agents HFBA and PFPA, respectively. However, this approach introduces a high risk of ion suppression, memory effects, and MS ion source contamination (Bonfiglio *et al.*, 1999), which we wanted to avoid, especially for high-throughput analyses.

**Fig. 4.**
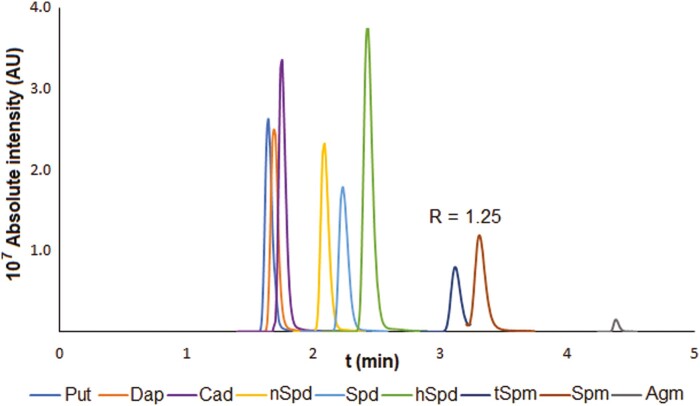
MRM chromatogram of benzoylated biogenic amines. R denotes the chromatographic resolution.

Our method was unable to separate the isomers tSpm and Spm, which have the same MRM transitions. tSpm is essential in biological processes, including vascular tissue development (Poidevin *et al.*, 2019) and plant defense (Marina *et al.*, 2013), and should, therefore, be quantified together with other biogenic amines. Because the method exhibited poor sensitivity for some underivatized biogenic amines and could not separate tSpm from Spm, the benzoylation strategy of Taibi *et al.* (2000) was adapted to facilitate the RP separation of biogenic amines. This enabled the successful separation of tSpm and Spm with a chromatographic resolution of R=1.25 (Fig. 4). It also increased the method’s sensitivity for other biogenic polyamines by up to three orders of magnitude. High sensitivity is very desirable in high-throughput analysis because it reduces the amount of plant tissue that is needed, so maximizing sensitivity was a key consideration during method development.

### Development and optimization of a plant tissue extraction protocol

Matrix effects can profoundly affect the extraction of amino acids and/or biogenic amines. Plant tissue is a very complex matrix containing both low molecular weight compounds and macromolecules with widely varying chemical structures and polarities. Before extraction, plant tissue must be homogenized to break the cell walls in the tissues and allow compounds of interest to migrate to the extraction solvent. Identifying a suitable extraction solvent is vital because the solvent strongly influences the extraction efficiency and stability of the target analytes. The most common solvents for extracting free amino acids from plant tissue are aqueous neutral or acidified solutions of ethanol or methanol (Bathurst, 1953; Thiele *et al.*, 2008), or even just water (Guo *et al.*, 2013; Zhou *et al.*, 2013), while biogenic amines are commonly extracted with aqueous solutions of trichloroacetic acid (TCA) or perchloric acid (Taibi *et al.*, 2000). However, these acids are unsuitable if the extracts are to undergo LC-MS/MS analysis because of their ion-suppressing and oxidizing properties; if their use is unavoidable, sample clean-up before analysis is strongly recommended.

Purification and pre-concentration via SPE is a common post-extraction step in methods for quantifying plant metabolites (Tarkowská *et al.*, 2014). The solid adsorbent in SPE cartridges either retains compounds of interest by separating them from matrix compounds or removes components that might interfere with the analysis, allowing them to pass through. QuECHeRS is an alternative quick, easy, cheap, effective, rugged, and safe purification technique that is widely used to purify samples of diverse matrices, including plant tissue (Rejczak and Tuzimski, 2015; Sarraf *et al.*, 2020). The advantages of QuEChERS include short sample preparation times and the use of small amounts of material and solvent.

Since this study focused on zwitterionic aliphatic amino acids as well as aliphatic, aromatic, and heterocyclic biogenic amines, we sought to identify the most general and effective combination of extraction solvent and purification technique for isolating these compounds from plant tissues. Lyophilized and pulverized Arabidopsis rosettes were used as a representative plant tissue matrix in these experiments. As shown in Fig. 1, six different extraction solvents and eight isolation protocols were tested. In addition to analyses of underivatized extracts, samples were analyzed after undergoing clean-up by SPE and QuEChERS to remove chlorophylls and other lipophilic compounds that were not of interest in this work and could cause significant matrix effects.

**Fig. 5.**
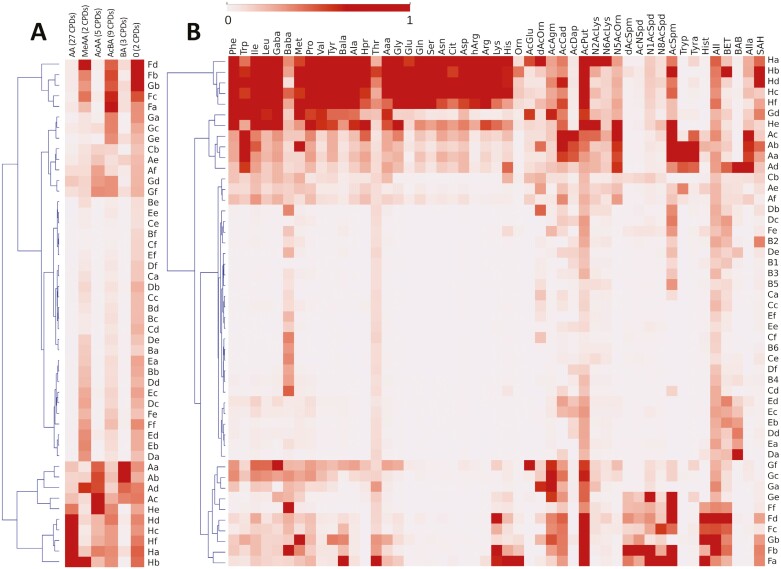
Extraction efficiencies of nitrogenous compounds in lyophilized Arabidopsis tissue. (A) Heatmap of the log ratios of the total amounts of amino acids (AA), methylated amino acids (MeAA), acetylated amino acids (AcAA), biogenic amines (BA), acetylated biogenic amines (AcBA), and urea derivatives (O). (B) Heatmap of the log ratios of individual analytes.

Two heatmaps were constructed to visualize the results of the extraction and purification study. Each extraction and purification protocol was named using a mixture of uppercase letters denoting the extraction protocol and lowercase letters denoting the extraction solvent (Fig. 5; Supplementary Dataset S1). The first heatmap (Fig. 5A) shows the results based on the measured total concentrations of different analyte groups (e.g. free amino acids, biogenic amines, and so on). The data were separated into two subclusters and clustered into two main groups, with the A and H extraction protocols being clearly separated from the others (see Fig. 1). The H protocols provided high extraction efficiencies for free amino acids, while the A protocols performed well for acetylated amino acids and biogenic amines. The A and H protocols also performed well for other nitrogen-containing metabolites. Conversely, the G protocols were more efficient for methylated compounds, acetylated biogenic amines, and others (Fig. 5A). To enable deeper analysis, a second heatmap was constructed, showing the log ratios for each analyte individually (Fig. 5B). As before, the extraction protocols in this heatmap were divided into two subclusters—one containing only the H protocols and the other containing all the other protocols. The H protocols had the highest extraction efficiencies when considering the full set of target analytes. The second subcluster was further subdivided into additional subclusters—the A protocols were widely separated from the F and G protocols, largely because of their differing efficiencies in the extraction of biogenic amines and acetylated and methylated compounds (Fig. 5). Extraction protocol H involved analyzing samples directly after extraction without purification, while the A protocol involved SPE clean-up with a universal hydrophilic–lipophilic-balanced (HLB) polymeric RP sorbent. Gao *et al.* (2016) previously used a cation exchange SPE sorbent to purify amino acid-containing aqueous extracts from soil samples, but this sorbent appeared to be unsuitable for the compounds examined here. Based on the findings of Šimura *et al.* (2018), samples processed using protocol A were passed through the HLB cartridge and eluted in flow-through mode. As a result, most of the amino acids remained on the sorbent, resulting in lower recoveries than for protocol H (Fig. 5).

Protocols F and G involved QuEChERS clean-up with a sorbent comprising 150 mg of MgSO_4_/25 mg of PSA/50 mg of C18 (protocol F) or 150 mg of MgSO_4_/50 mg of C18 (protocol G). While PSA (primary–secondary amine sorbent) is a weak cation exchanger and mainly absorbs organic acids, C18 mainly absorbs lipophilic compounds. Although these sorbents both provided good selectivity for certain biogenic amines and their acetylated derivatives, their selectivity and retention of amino acids were unsatisfactory. The QuEChERS sorbents used in protocols B, C, D, and E contained GCB (graphitized carbon black), and achieved even worse selectivity, suggesting that GCB adsorbed many compounds of interest. Similar outcomes were reported by Dong and Xiao (2017), who found that the best selectivity for aliphatic biogenic amines was achieved using a mixture of PSA and C18 sorbents.

**Fig. 6.**
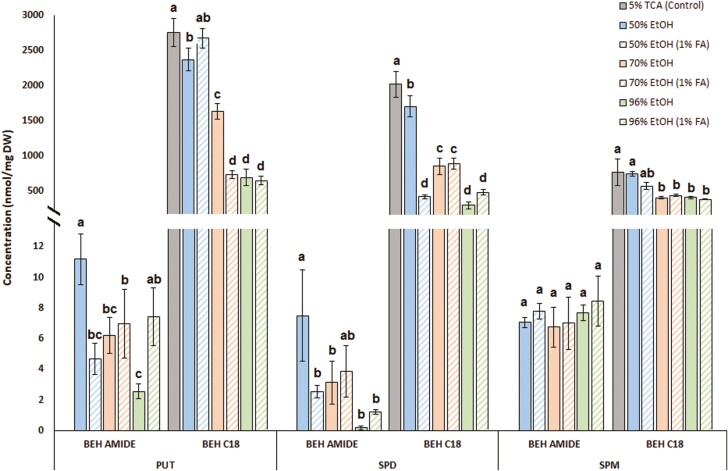
Extraction efficiencies and sensitivities for selected aliphatic biogenic amines analyzed on the BEH AMIDE column and after derivatization on the BEH C18 column. Values for the same extraction solvent labeled with the same letter do not differ significantly according to ANOVA with post-hoc Newman–Keuls analysis at a significance level of *P*<0.05. All measurements were performed on three biological replicates.

The choice of extraction solvent also strongly influenced the extraction efficiency for all analyte groups (Fig. 3). We therefore compared the extraction efficiencies achieved with the solvents listed in the Materials and methods with that achieved with 5% TCA, which is a conventional solvent for isolating aliphatic biogenic amines. As shown in Fig. 6, the highest efficiencies were obtained using 50% EtOH (solvent 1) and 50% EtOH acidified with 1% FA (solvent 2). The good performance of solvent 2 is consistent with the common use of FA to increase extraction and ionization efficiency in positive mode ESI (Su *et al.*, 2021). The results presented in Fig. 6 also show that derivatization of aliphatic biogenic amines followed by separation on an RP column improved the method’s sensitivity by a few orders of magnitude when compared with direct analysis of underivatized compounds on the HILIC column. While 5% TCA was the best solvent for extracting biogenic amines, its efficiency was comparable with that achieved by extraction with neutral 50% EtOH (solvent 1) followed by derivatization. Solvent 1 was, therefore, used to extract target analytes in all subsequent experiments.

**Fig. 7.**
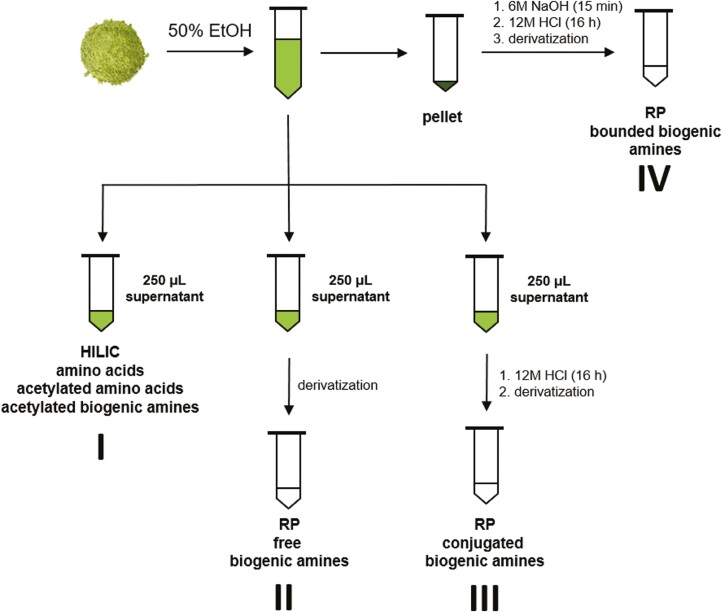
Extraction protocol for isolation of free amino acids, acetylated amino acids and biogenic amines, free biogenic amines, and conjugated and bound biogenic amines from a single plant extract.

When extracting 2–5 mg of lyophilized plant tissue with 1 ml of 50% EtOH, only a 200 μl aliquot of the extract was needed to quantify nitrogenous plant metabolites using HILIC, making it possible to use a second 200 μl aliquot to quantify free aliphatic biogenic amines after derivatization and RP separation. However, aliphatic amines in plant tissues are present as free species but also in conjugated and bound forms, both of which play vital roles in plant development and abiotic and biotic stress responses (Bagni and Tassoni, 2001; Gonzalez *et al.*, 2021; Pal *et al.*, 2021). The ability to monitor changes in their abundance could thus provide new insights into their roles in plant growth and stress tolerance. A slightly altered version of the method reported by Piqueras *et al.* (2002) was therefore applied to the remaining 600 μl of the extract and the pellet left after centrifugation to determine the tissue sample’s conjugated and bound biogenic amine content. Biogenic amines are typically conjugated with small molecules such as amides or hydroxycinnamic acids (Bagni and Tassoni, 2001) and can be quantified in their free forms after acid hydrolysis. However, bound biogenic amines are bonded to macromolecules such as nucleic acids, proteins, or lipids. To liberate these bound species, they must be hydrolyzed first with a strong base and then with acid, as shown in Fig. 7 before they can be quantified.

### Method validation

The method’s overall selectivity was evaluated by assessing its chromatographic and mass spectrometric selectivity separately. Chromatographic selectivity was evaluated by checking that the naturally occurring analytes co-eluted with the corresponding authentic standards and testing retention time stability (Supplementary Tables S1, S2). Importantly, isomers with the same MRM transitions were generally adequately separated with resolution factors >1.5 (Fig. 3); the only exceptions were tSpm and Spm, for which the resolution factor was 1.25 (Fig. 4). Mass spectrometric selectivity was evaluated by examining analytes with multiple MRM transitions to verify the method’s ability to distinguish between compounds with identical chromatographic retention times and molecular masses but different structures. Sixty of the target analytes had at least three characteristic MRM transitions, but 14 had only two (Supplementary Tables S1, S2) because the limited intensity of some MS fragments made it impossible to identify a third MRM transition for all analytes.

Calibration curves were established based on 10 calibration points. Their linearity was significantly improved by logarithmic transformation, which maximizes the detection range (and thus increases sensitivity) without reducing accuracy (Kong *et al*., 2021). The linear and logarithmic regression curves for selected analytes are shown in Table 1, clearly demonstrating that the transformation improves the curves’ regression coefficients and linear ranges. Other authors who used linear regression ranges obtained relatively narrow quantification ranges similar to those presented here (Guo *et al.*, 2013; Zhou *et al.*, 2013).

**Table 1.** Linear and logarithmic regression curve properties for selected analytes

Compound	Linear regression			Logarithmic regression		
	Equation	*R* ^2^	Range (μmol l^–1^)	Equation	*R* ^2^	Range (μmol l^–1^)
Leu	*y*=1.0941*x*–0.1359	0.9802	1–50	log *y*=0.9973(log *x*)–0.0702	0.9965	0.01–50
GABA	*y*=1.9767*x*–0.0817	0.9942	1–50	log *y*=1.0058(log *x*)+0.2325	0.9977	0.01–50
Pro	*y*=0.1640*x*–0.0059	0.9941	1–50	log *y*=0.9198(log *x*)–0.6700	0.9952	0.01–50
Thr	*y*=0.7394*x*–0.0918	0.9899	1–50	log *y*=1.1012(log *x*)–1.2271	0.9980	0.1–50
Glu	*y*=2.0740*x*–0.1162	0.9935	1–50	log *y*=0.9798(log *x*)+0.3161	0.9970	0.05–50
Gln	*y*=2.1113*x*–0.1680	0.9838	1–50	log *y*=1.0163(log *x*)+0.2260	0.9977	0.025–50
Dap	*y*=0.2610*x*+0.0352	0.9489	1–25	log *y*=1.0228(log *x*)–0.3059	0.9974	0.01–25

The validation data for the compounds analyzed in HILIC mode are summarized in Table 2; the corresponding data for compounds analyzed after RP separation are summarized in Table 3. Both tables include logarithmic ranges, LODs, LOQs, and the intra- and inter-day precision. Precision was quantified in terms of the RSD (%) for low, intermediate, and high-concentration quality control (QC) samples.

**Table 2.** Method validation parameters for the compounds analyzed on the BEH AMIDE column

Compound	Range (μmol l^–l^)	LOD (fmol per injection)	LOQ (fmol per injection)	Intra-day precision (RSD %)			Inter-day precision (RSD %)		
				Low QC	Mid QC	High QC	Low QC	Mid QC	High QC
AcPEA	0.01–50	4.8	15.9	17.01	17.55	6.75	13.54	15.30	5.98
AcGABA	0.01–25	8.1	27.0	6.21	8.34	9.09	9.80	7.77	6.43
AcBAla	0.01–25	12.2	40.5	2.24	7.93	5.58	5.58	6.99	5.09
dAcLys	0.01–50	3.0	10.0	5.34	4.76	7.07	14.86	4.18	16.67
AcGlu	0.025–50	13.6	45.4	11.90	17.57	7.86	12.32	17.02	13.54
dAcOrn	0.025–50	24.9	83.0	4.33	6.40	1.35	4.85	13.91	6.78
N2AcLys	0.01–50	100.2	333.9	5.16	9.07	4.45	8.01	7.72	16.79
N2AcOrn	0.01–50	209.5	698.2	7.92	8.84	8.63	11.26	8.32	15.20
N6AcLys	0.01–50	69.6	232.1	16.60	6.49	6.05	11.94	14.16	15.41
N5AcOrn	0.01–50	210.5	701.7	10.29	7.03	2.36	8.76	11.54	9.65
AcCit	0.01–50	310.4	1034.8	9.83	10.40	2.06	12.86	10.95	2.70
AcTryp	0.01–50	6.2	20.7	10.52	7.03	8.59	8.41	7.54	6.33
AcTyra	0.01–50	1.6	5.4	7.46	5.74	5.27	10.88	4.99	18.97
dAcDap	0.01–5	14.8	49.4	13.73	4.57	10.26	17.22	8.96	9.11
AcAgm	0.01–25	24.4	81.4	16.87	13.30	5.98	16.81	10.15	4.23
AcHist	0.01–25	5.5	18.5	11.60	9.01	1.86	9.12	12.86	3.96
AcCad	0.01–50	3.5	11.8	6.75	17.37	4.30	6.42	13.87	3.28
AcDap	0.01–50	64.1	213.6	8.38	8.03	3.78	7.32	8.77	9.79
AcPut	0.01–50	232.9	776.4	5.78	2.96	3.17	7.10	5.01	4.73
dAcSpm	0.01–50	21.0	70.0	13.17	14.68	2.89	10.95	10.59	6.36
N1AcSpd	0.01–100	31.4	104.8	13.20	9.72	14.67	16.89	12.81	11.15
AcNSpd	0.025–50	20.2	67.4	15.79	5.98	4.48	11.61	4.85	12.88
N8AcSpd	0.025–100	659.6	2198.8	1.91	16.11	5.56	8.54	15.41	5.55
N1AcSpm	0.025–25	816.1	2720.3	2.83	3.02	13.23	8.25	5.78	16.56
Tryp	0.01–100	9.4	31.3	6.95	7.98	5.42	12.35	10.85	11.69
Tyra	0.025–250	88.0	293.3	12.31	12.42	1.76	19.59	11.59	1.82
Hist	0.01–250	59.2	197.5	4.65	5.78	9.12	9.14	6.85	10.25
Pglu	0.025–250	5.9	24.7	11.46	9.80	2.16	14.87	9.95	4.30
BAB	0.01–100	73.9	246.3	7.86	6.60	7.66	6.83	13.83	11.20
Leu	0.01–250	256.6	855.3	6.58	7.60	3.20	11.98	12.48	5.81
Phe	0.025–250	68.0	226.7	2.99	14.17	3.38	3.42	10.55	2.88
Trp	0.025–250	378.5	1261.5	3.73	2.14	6.14	7.10	5.01	4.73
GABA	0.01–250	161	536.5	3.22	3.09	15.81	2.46	9.99	12.14
Ile	0.01–250	177.1	590.3	3.57	2.18	3.74	4.56	4.65	17.48
Sta	0.05–250	71.2	236.8	12.04	7.90	6.95	12.97	11.60	8.35
GB	0.05–250	281.7	939.0	9.22	11.72	5.49	11.08	15.35	7.61
BABA	0.025–100	934	3113.3	3.23	7.71	6.71	8.76	8.82	6.14
Met	0.025–250	415.7	1385.6	12.82	5.81	0.95	18.02	4.58	3.52
Val	0.025–250	165.1	550.4	3.92	3.56	8.24	3.44	9.51	6.23
hCys	0.05–250	748.3	2494.3	7.28	7.16	0.84	9.78	5.14	1.26
Pro	0.01–100	163.3	544.4	9.80	5.27	2.68	15.15	3.83	2.08
BAla	0.025–100	356.2	1187.2	7.01	9.36	7.39	5.60	8.26	9.68
Cys	0.5–250	5128.2	17 094	4.21	13.99	12.18	4.58	13.22	13.78
Tyr	0.025–250	163.6	545.4	3.05	5.13	5.04	3.28	3.68	4.12
Ala	0.025–250	642.1	2140.4	12.10	1.32	1.94	17.88	12.31	3.06
Hyp	0.05–250	1781.5	5938.2	12.49	10.60	3.21	10.43	7.94	6.91
Aaa	0.025–250	331.7	1105.6	18.13	10.94	3.73	16.11	7.93	7.42
Thr	0.1–250	55.4	184.7	9.19	4.73	3.46	11.64	10.99	5.54
Gly	0.25–250	4411.8	14705.9	7.67	3.86	9.05	8.91	5.48	8.26
Glu	0.05–250	472.4	1574.6	10.58	9.42	6.43	8.61	16.05	14.24
Gln	0.025–250	194.8	649.3	10.38	8.73	3.18	7.57	10.46	14.49
Ser	0.05–250	1310.6	4368.7	9.01	1.83	6.13	12.47	5.57	6.48
Asp	0.1–100	23006.1	76687.1	4.33	1.13	2.69	4.37	7.11	18.03
Asn	0.05–250	1541.6	5138.7	11.01	6.24	5.20	7.80	12.37	8.52
Cit	0.05–250	97.7	325.8	9.87	6.31	2.79	8.08	7.31	14.65
SAH	0.025–250	74.1	246.9	11.60	10.14	10.24	19.70	8.93	11.28
hArg	0.05–250	383.4	1278.1	4.64	3.93	6.46	11.19	16.14	5.26
Arg	0.05–250	192.4	641.4	9.18	6.12	17.28	13.06	6.24	19.95
His	0.1–250	958.8	3195.9	9.73	6.89	8.92	19.06	6.41	9.57
Lys	0.05–250	469.4	1564.7	3.84	9.56	9.08	5.53	7.37	7.98
Orn	0.05–250	299.9	999.5	6.39	6.79	6.46	9.98	7.68	17.32
SAM	0.025–100	6526.0	21753.3	15.24	19.35	3.96	15.85	17.64	15.85
Cis	0.05–250	5348.5	17828.5	5.04	5.14	4.10	12.18	11.22	5.83
All	0.01–250	2.5	8.4	11.29	8.06	3.26	11.71	7.11	11.19
Alla	0.05–100	10.8	36.6	5.75	4.79	8.11	5.54	3.40	6.82

**Table 3.** Method validation parameters for aliphatic biogenic amines analyzed on the BEH C18 column

Compound	Range (μmol l^–1^)	LOD (fmol per injection)	LOQ (fmol per injection)	Intra-day precision (RSD %)			Inter-day precision (RSD %)		
				Low QC	Mid QC	High QC	Low QC	Mid QC	High QC
Put	0.005–25	23.2	77.2	2.44	1.83	2.58	3.10	1.93	4.04
Dap	0.005–25	28.4	94.8	2.90	2.65	2.54	12.67	12.60	3.75
Cad	0.005–25	21.2	70.7	3.99	0.42	1.02	21.31	9.61	4.58
nSpd	0.005–25	21.9	72.8	N.A.	2.82	1.36	N.A.	11.31	4.92
Spd	0.005–25	20.9	69.6	2.78	0.97	3.42	4.21	1.81	4.82
hSpd	0.005–25	34.5	114.9	2.02	2.13	1.05	10.42	4.84	5.14
tSpm	0.025–25	109.4	364.8	4.12	2.98	1.19	4.96	3.45	4.44
Spm	0.025–25	95.7	319.0	2.37	2.37	1.39	12.40	12.90	9.27
Agm	0.05–25	405.1	1350.5	6.13	12.06	6.73	15.60	10.76	13.17

NA, not available.

The calibration curves for each analyte indicated wider analytical ranges than those previously reported in the literature (Guo *et al.*, 2013; Gao *et al.*, 2016; Wen *et al.*, 2019; Su *et al.*, 2021). The LODs and LOQs varied greatly depending on the chemical structure of the analyte. In general, the highest sensitivities were achieved for acetylated amino acids and biogenic amines (Table 2). The intra-day RSD based on three independent injections at each of the low, intermediate, and high concentration levels was <20%, indicating good intra-day precision. Similar results were obtained in inter-day precision measurements where samples were analyzed every second day over a 6 d period (Tables 2, 3) and stored in an autosampler at 4 °C between analyses. Inter-day precision was evaluated over a 6 d period rather than over three consecutive days because metabolomic studies of plant tissues typically require the analysis of hundreds of samples. Analyte stability was also investigated, revealing that underivatized analytes separated on the HILIC column exhibited acceptable stability (<5% decomposition after 48 h) while derivatized biogenic amines exhibited ~30% decomposition over the same period. However, the rate of decomposition observed for the ISs is comparable with that for the derivatized extracts. Therefore, the decomposition would not affect the calculated analyte levels.

Process efficiencies and matrix effects for analytes that had deuterated analogs were calculated using those analogs as standards (Table 4). For other analytes, these effects were calculated in a similar manner using the isotopically labeled IS that most closely resembled the analyte of interest in terms of structure, method sensitivity, and chromatographic behavior. Su *et al.* (2021) and Wen *et al.* (2019) used a similar approach: Su *et al.* (2021) used data for two amino acids and one biogenic amine to quantify 14 different metabolites belonging to the urea cycle, including acetylated derivatives of biogenic amines, while Wen *et al.* (2019) used three internal standards to quantify 19 amino acids. Although we did not use every commercially available isotopically labeled analog of the target analytes, the ISs that we did use represented every compound type of interest in this work. Thus, d_6_-dAcDap was used to quantify AcPEA, AcTryp, AcTyra, AcGABA, AcBAla, dAcDap, Pglu (l-pyroglutamic acid), dAcLys (*N*2,*N*6-diacetyllysine), AcGlu (*N*-acetylglutamic acid), and dAcOrn (*N*2,*N*5-diacetylornithine); d_2_-AcDap (*N*-acetyldiaminopropane-1,1-d_2_) was used to quantify Tryp (tryptamine), All, AcCit (*N*2-acetylcitrulline), Tyra (tyramine), AcAgm (*N*4-acetylagmatine), AcHist (*N*-acetylhistamine), AcCad (*N*-acetylcadaverine), AcDap (*N*-acetyldiaminopropane), and AcPut (*N*-acetylputrescine); d_10_-Leu (leucine-2,3,3,4,5,5,5,5ʹ,5ʹ,5ʹ-d_10_) was used to quantify BAB (β-alanine betaine), Leu (leucine), Phe (phenylalanine), Trp (tryptophan), and Ile (isoleucine); d_6_-GABA (γ-aminobutyric acid-2,2,3,3,4,4-d_6_) was used to quantify GABA (γ-aminobutyric acid), Sta (stachydrine), GB (glycine betaine), BABA (β-aminobutyric acid), and Met (methionine); ^13^C_5_,^15^N-Pro (proline-^13^C_5_,^15^N) was used to quantify hCys (homocysteine), Pro (proline), Val (valine), Cys, Tyr (tyrosine), and Alla; d_4_-Ala (alanine-2,3,3,3-d_4_) was used to quantify Bala (β-alanine), N2AcLys (*N*2-acetyllysine), Ala (alanine), N2AcOrn (*N*2-acetylornithine), Hyp (*trans*-2-hydroxyproline), AAA (2-aminoadipic acid), N6AcLys (*N*6-acetyllysine), and N5AcOrn (*N*5-acetylornithine); ^13^C_4_,^15^N,d_5_-Thr (threonine-^13^C_4_,^15^N,2,3,4,4,4-d_5_) was used to quantify Thr (threonine) and Gly (glycine); d_5_-Glu (glutamic acid-2,3,3,4,4-d_5_) was used to quantify Glu (glutamic acid); d_5_-Gln (glutamine-2,3,3,4,4-d_5_) was used to quantify Gln (glutamine), Ser (serine), Asp (aspartic acid), Asn (asparagine), Cit (citruline), SAH (*S*-adenosylhomocysteine), hArg (homoarginine), Arg (argininge), His (histidine), Hist (histamine), Lys, Orn (ornithine), SAM [*S*-(5ʹ-adenosyl)-l-methionine], and Cis (cistine); d_6_-N1AcSpd was used to quantify dAcSpm, N1AcSpd (*N*1-acetylspermidine), AcNSpd, and N8AcSpd (*N*8-acetylspermidine); and, finally, d_6_-N1AcSpm was used for to quantify N1AcSpm (*N*1-acetylspermine). The aliphatic biogenic amines Put, Dap (diaminopropane), Cad, nSpd, Spd, hSpd, tSpm, Spm, and Agm were quantified using the RP pre-column derivatization method with d_6_-Dap as the labeled standard.

**Table 4.** Process efficiency (PE) and matrix effects (ME) for different plants analyzed on BEH AMIDE and BEH C18 columns.

Plant	Effect	Leu	Pro	Ala	Thr	Glu	Gln	dAcDap	AcDap	N1AcSpd	N1AcSpm	Dap^a^
Radish	PE	68.8 ± 0.9	32.7 ± 2.7	49.3 ± 2.4	51.5 ± 3.6	27.9 ± 2.0	34.3 ± 8.0	45.8 ± 4.1	49.7 ± 3.7	18.3 ± 6.9	16.2 ± 4.2	59.5 ± 4.5
	ME	74.6 ± 4.3	49.6 ± 4.9	50.7 ± 4.8	56.4 ± 3.8	33.1 ± 3.8	38.8 ± 1.5	50.3 ± 6.8	47.6 ± 6.0	20.0 ± 4.4	19.4 ± 3.6	78.9 ± 5.5
Maize	PE	36.0 ± 1.3	46.0 ± 1.5	42.8 ± 3.2	44.3 ± 6.7	31.4 ± 3.7	32.7 ± 1.5	45.3 ± 1.3	33.9 ± 2.7	21.0 ± 2.1	19.4 ± 3.1	72.0 ± 6.0
	ME	45.1 ± 6.0	48.5 ± 1.3	45.1 ± 3.3	49.5 ± 3.6	33.8 ± 8.7	36.6 ± 0.7	55.8 ± 0.3	36.9 ± 1.8	22.1 ± 1.5	22.3 ± 4.1	73.8 ± 4.3
Barley	PE	56.7 ± 9.0	54.1 ± 5.0	61.4 ± 6.1	52.3 ± 6.2	34.6 ± 9.0	34.1 ± 1.6	50.7 ± 4.1	41.5 ± 2.3	24.5 ± 4.9	18.2 ± 4.1	69.0 ± 8.3
	ME	59.9 ± 7.2	55.0 ± 3.7	70.7 ± 12.3	56.7 ± 7.5	46.3 ± 6.5	43.4 ± 9.2	49.1 ± 8.7	47.1 ± 5.9	26.2 ± 7.9	21.3 ± 1.9	74.3 ± 5.2
Wheat	PE	50.0 ± 9.2	41.0 ± 4.4	50.7 ± 21.8	51.3 ± 3.7	31.8 ± 7.7	31.1 ± 3.5	55.4 ± 2.5	45.1 ± 7.5	19.2 ± 8.1	20.3 ± 4.1	67.2 ± 3.6
	ME	53.0 ± 5.4	43.6 ± 3.9	55.4 ± 10.7	55.4 ± 5.9	36.4 ± 5.5	39.9 ± 5.7	57.7 ± 5.4	44.3 ± 9.1	26.5 ± 4.0	22.7 ± 1.9	78.0 ± 4.5
Tomato	PE	55.5 ± 5.2	46.1 ± 2.5	50.6 ± 4.8	49.5 ± 4.6	37.3 ± 7.9	39.9 ± 7.8	53.0 ± 1.3	49.8 ± 7.5	21.9 ± 12.1	19.3 ± 2.7	57.1 ± 3.7
	ME	52.6 ± 0.4	51.8 ± 6.6	57.3 ± 11.9	50.3 ± 6.9	43.4 ± 7.0	40.6 ± 3.1	56.2 ± 4.7	49.1 ± 5.4	25.9 ± 5.2	19.6 ± 3.1	67.8 ± 8.3
Tobacco	PE	47.1 ± 2.1	41.4 ± 2.3	50.7 ± 5.6	44.2 ± 4.6	27.2 ± 9.7	31.7 ± 1.5	53.7 ± 6.3	50.7 ± 7.8	22.3 ± 0.4	19.7 ± 3.8	62.9 ± 5.6
	ME	46.4 ± 2.1	45.9 ± 6.7	51.5 ± 4.7	46.9 ± 8.1	33.5 ± 2.0	37.9 ± 9.7	56.0 ± 3.6	51.3 ± 3.8	28.4 ± 4.1	21.2 ± 4.3	68.0 ± 4.4
Arabidopsis	PE	54.9 ± 5.0	53.6 ± 6.3	65.2 ± 7.5	65.1 ± 5.3	38.7 ± 12.6	35.4 ± 9.1	53.2 ± 2.0	75.6 ± 4.5	32.6 ± 4.5	26.3 ± 4.3	63.1 ± 8.2
	ME	67.6 ± 6.2	58.4 ± 2.4	67.0 ± 6.2	68.4 ± 3.5	46.3 ± 7.6	40.3 ± 5.6	61.2 ± 7.9	77.9 ± 6.4	34.2 ± 2.3	27.9 ± 4.3	75.3 ± 5.1

Results are presented as mean values (±SD) of three individual biological replicates.

^a^ Dap was analyzed on the BEH C18 column.

Process efficiency and matrix effects were investigated by adding the relevant IS at a concentration of 5 μM for HILIC or 1 μM for RP mode at the beginning or end of the isolation protocols, respectively. These effects were investigated in seven plant species: Arabidopsis, radish, maize, barley, wheat, tomato, and tobacco (Table 4). Three biological replicates were used for each plant and the results obtained are presented as mean values (±SD).

Based on the results presented in Table 4, this method has a strong matrix effect that causes significant ion signal suppression, especially at the end of the elution gradient, where N1AcSpd and N1AcSpm elute. Such effects are typical for samples of biological origin, particularly plant tissue samples, when analyzed without sample clean-up, and can be overcome with labeled ISs (Smeraglia *et al.*, 2002). However, there were no significant differences between matrix effects (ME) and the process efficiency (PE) values obtained for any of the seven plant species when analyzed via HILIC, meaning that the extraction recovery of the target analytes was close to 100% (Matuszewski *et al.*, 2003). For aliphatic biogenic amines analyzed by RP after extraction and derivatization, the differences between ME and PE were at most 10%, meaning that these processes slightly reduced recovery. However, RP mode analysis increased the PE and ME for these compounds overall because derivatization and extraction after derivatization constitutes a form of sample clean-up and thus reduces ion suppression.

Carryover was tested by injecting blank solvent directly after injecting a high concentration calibration curve sample; in all cases, the chromatograms for the solvent blank showed no peaks corresponding to any monitored MRM transition.

### Characterization of diverse plant tissue matrices using the developed method

**Fig. 8.**
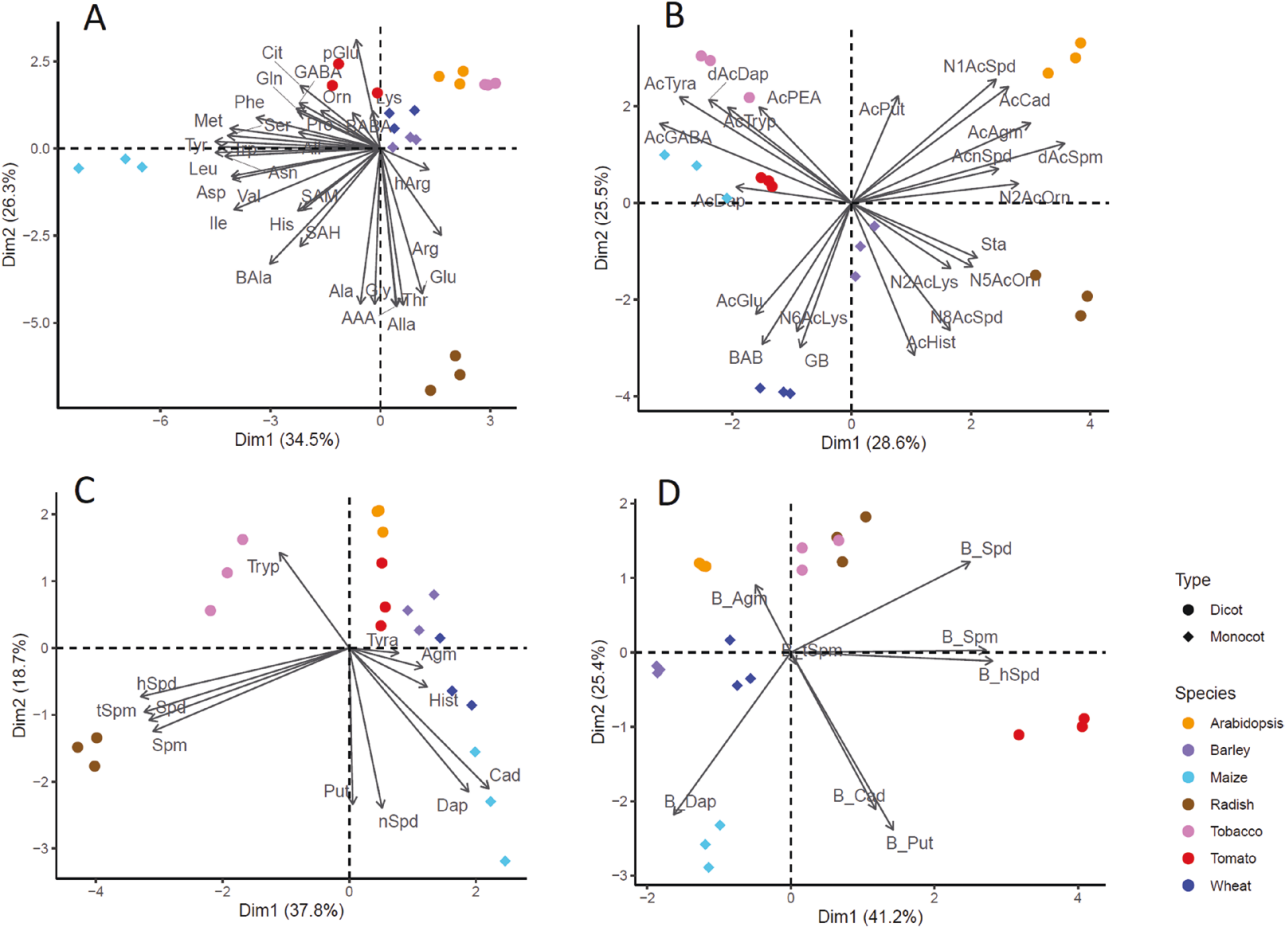
Principal component (PC) analysis of changes in nitrogen-related metabolite levels within seedlings of seven plant species. (A) Free amino acids and other nitrogen-related metabolites. (B) Acetylated and methylated polyamines and amino acids. (C) Free biogenic amines. (D) Bound biogenic amines. Dim1 and Dim2 denote PC1 and PC2, respectively.

The proposed method was used to analyze nitrogenous plant metabolites in seedlings of seven different plant species, some of which are frequently used as models in plant science research. Extraction was performed with the protocol described in Fig. 7 using three biological replicates of each plant species (Supplementary Dataset S2). PCA was used on four of the resulting datasets to visualize the differences between the species (Fig. 8). In the first PCA, which examined the endogenous levels of free amino acids and other nitrogen-containing metabolites, the first principal component (PC), Dim1, accounted for 34.5% of the total variance and mainly separated maize seedlings from the other plant species due to their high content of specific amino acids, namely Met, Tyr, Leu, Asp, and Trp (Fig. 8A). The second PC, Dim2, explained 26.3% of the variance in the data and indicated that radish seedlings were associated with the amino acids Arg, Glu, AAA, Thr, Ala, and Gly, as well as the nitrogen-containing metabolite Alla. However, they had the lowest levels of pGlu, GABA, Orn, Cit, and Lys, which was more abundant in all other species, but especially in tomato.

The second PCA examined levels of acetylated and methylated nitrogen compounds (Fig. 8B), and separated the seven species into four distinct groups along its first two PCs, Dim1 and Dim2. The biggest group consisted of tomato, tobacco, and maize, and was associated with higher levels of AcDap, dAcDap, AcTryp, AcTyra, and AcPEA. The second group, consisting of Arabidopsis, had the highest levels of N1AcSpd, AcCad, AcAgm, AcnSpd, and dAcSpm at the studied developmental stage. The third group comprised radishes, which had high levels of N5AcOrn, N2AcLys, N8AcSpd, AcHis, and Sta. The final group consisted of wheat seedlings, which exhibited high levels of AcGlu, and N6AcLys, as well as the methylated amino acids BAB and GB. Interest in acetylated amino acids and biogenic amines has increased in recent years, and it has been shown that the abundance of different amino acid forms can vary among species (Bělíček *et al.*, 2023). However, their biological relevance in plants still needs to be determined. Some evidence supporting biological roles for these substances exists; for example, Abdelhakim *et al.* (2022) observed that AcGlu accumulated in wheat plants mainly when grown under optimal conditions but became less abundant during stress responses. They also found that its abundance correlated positively with photosynthesis and final yields. Additionally, the accumulation of acetylated Dap forms has been linked to stomatal closure and plant defense against stress (Jammes *et al.*, 2014; Hernándiz *et al.*, 2022a; Gulenturk *et al.*, 2023).

Methylated amino acids, which are usually known as betaines, are quaternary ammonium compounds that mainly accumulate under adverse conditions and are considered essential markers of plant stress tolerance against different stressors (Ashraf and Harris, 2004). BAB is thus considered an osmoprotectant and may be more effective in this role than GB (reviewed by Parthasarathy *et al.*, 2019), suggesting that wheat is one of the major crops that is best able to deal with stress conditions. Our results clearly show that the levels of acetylated and methylated amino acids differ markedly between species and could thus be useful for chemical characterization of not only species but also genotypes during selection programs.

The final two PCAs examined different biogenic amine fractions (Fig. 8C, D). The first examined free polyamines and once again clearly separated the seven plant species: barley, wheat, and tomato seedlings had the highest Tyra, Hist, and Agm levels; maize was associated with high levels of Cad, Dap, nSpd, and Put; tobacco had the highest levels of Tryp; and radish seedlings had the greatest concentrations of longer biogenic amines such as hSpd, Spd, Spm, and tSpm (Fig. 8C). Among the common biogenic amines, Put and Spd are essential for life and, together with Spm and Tspm, are involved in plant defense against abiotic and biotic stresses (Gonzalez *et al.*, 2021). However, the balance among them differs between species. Spm was reported to be the most efficient polyamine for improving plant defense (reviewed by Seifi and Shelp, 2019). However, treatment with exogenous Put and Spd enhanced drought tolerance and increased final yields in maize (Hernándiz *et al.*, 2022b). Little is known about the biological functions of uncommon biogenic amines such as Cad, nSpd, Tyra, and Tryp in plants. However, all of them are products of amino acid catabolism and are accumulated strongly under stress conditions (Marchetti *et al.*, 2019). They are also precursors of other compounds involved in plant defense or relevant for specific plant developmental stages. For example, Tryp is the entry point to metabolic routes leading to synthesizing indole alkaloids, including serotonin and melatonin (Negri *et al.*, 2021). Conversely, Tyra is formed by tyrosine decarboxylation catalyzed by tyrosine decarboxylase (TYDC; EC 4.1.1.25) and is subsequently hydroxylated by monophenol hydroxylase (MH) to form dopamine, whose accumulation enhances plant growth and stress resilience (reviewed by Liu *et al.*, 2020).

The final PCA focused on bound biogenic amines, revealing clear differences in their abundance within the studied species (Fig. 8D). Arabidopsis seedlings had the highest contents of bound Agm but lower levels of Cad and Put, like tobacco and radish. Maize had the highest levels of bound Dap, while tomato accumulated more bound Spm and hSpd (Fig. 8D). There is evidence that bound biogenic amines are involved in transcriptional and post-transcriptional regulation in plant cells (Poidevin *et al.*, 2019; Jiménez-Bremont *et al.*, 2022).

Interestingly, conjugated biogenic amines were less abundant than their bound forms in these young seedlings (Supplementary Dataset S2); their levels were below the limits of detection in barley and maize. The only detectable conjugated polyamine in tomato seedlings was Dap, while tobacco contained conjugated nSpd. The highest levels and greatest numbers of conjugated biogenic amines were found in Arabidopsis and radish seedlings, with Spd being the most abundant (Supplementary Dataset S2). High levels of conjugated Spd and Spm in Arabidopsis were also reported by Hernandiz *et al.* (2022a). The main conjugated biogenic amines appear to be phenolamides, which are formed by reactions between hydroxycinnamic acid derivatives and aliphatic or aromatic amines. Mono- and di-putrescine, spermidine, and spermine phenolamides are thought to play roles in plant development and stress responses (Roumani *et al.*, 2021).

**Fig. 9.**
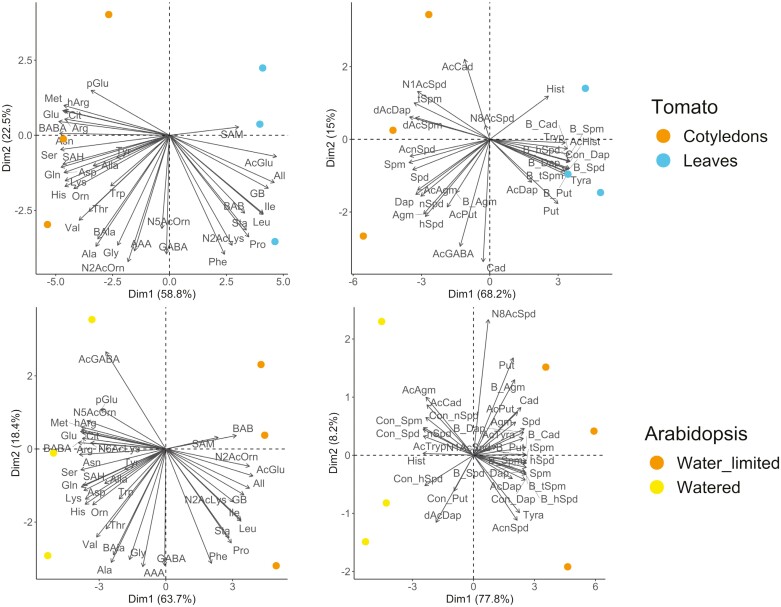
Principal component (PC) analysis of changes in nitrogen-related metabolite levels in tomato and Arabidopsis plants. (A) Free amino acids, other nitrogen-related metabolites, and acetylated and methylated amino acids in 15-day-old tomato seedlings with cotyledons only (red) and with true leaves (pink). (B) Free, conjugated, and bound biogenic amines in 15-day-old tomato seedlings with cotyledons only (red) and with true leaves (pink). (C) Free amino acids, other nitrogen-related metabolites, and acetylated and methylated amino acids in 4-week-old Arabidopsis under well-watered (yellow) and drought (orange) conditions. (D) Free, conjugated, and bound biogenic amines in 4-week-old Arabidopsis under well-watered (yellow) and drought (orange) conditions. Dim1 and Dim2 denote PC1 and PC2, respectively.

Two additional studies were performed using the developed method. The first compared the metabolic profiles of 15-day-old tomatoes with only cotyledons with those of seedlings whose first true leaves had formed, while the second compared 4-week-old Arabidopsis plants under well-watered and drought stress conditions (Fig. 9; Supplementary Dataset S3). In both cases, the studied treatments were well separated along the first PC. Tomato seedlings with true leaves had higher levels of AcGlu, SAM, All, GB, BAB, and Pro than seedlings showing only cotyledons (Fig. 9A). The high levels of AcGlu could be related to the higher photosynthetic activity of true leaves, in accordance with the preceding discussion, while the elevated levels of All, GB, BAB, and Pro may be related to a stronger antioxidative response in seedlings with true leaves, as has been observed in certain plant species (Wang *et al.*, 2019). Tomato seedlings with true leaves also had higher levels of bound and conjugated biogenic amines than those with only cotyledons (Fig. 9B). Conversely, seedlings without true leaves had higher levels of free and acetylated biogenic amines. This may be because the ongoing development of their true leaves required precise transcriptional and post-transcriptional regulation. Conversely, the cotyledons would have finished their development and entered senescence, which is associated with the accumulation of free biogenic amines such as Put, Spd, and Spm (Sobieszczuk-Nowicka *et al.*, 2016).

Differences were also observed in the nitrogen-containing metabolite profiles of well-watered versus water-limited Arabidopsis plants (Fig. 9C, D; Supplementary Dataset S3). It is well known that environmental stressors such as drought, salt stress, variations in nutrition, light intensity and quality, and temperature, among others, significantly alter plant metabolism (Annunziata *et al.*, 2017, 2018; Hernándiz *et al.*, 2022a; Ľuptáková *et al.*, 2023). Specifically, water restriction led to the accumulation of free amino acids as an osmotic response to maintain water status of plants under limited supply conditions (De Diego *et al.*, 2013). In line with this, drought stress resulted in an increased accumulation of Pro, and other antioxidative compounds such as the betaines GB, BAB, pGlu, and Sta, as well as free and bound biogenic amines including hSpd, Spd, Spm, and tSpm. Interestingly, the total amino acid pool was lower than expected, being even more reduced in the stressed plants than in controls (Supplementary Dataset S3). This could be due to the developmental stage of the analyzed plants, as amino acid levels decreased in Arabidopsis during later stages, particularly at the onset of the reproductive phase (Olas *et al.*, 2021). In contrast, Pro could be accumulated in older Arabidopsis plants, especially in reproductive organs (Chiang and Dandekar, 1995), aiding rapid inflorescence stem elongation, accelerating flowering, and enhancing fertility (Matiolli *et al.*, 2009; Funck *et al.*, 2012).

The absence of additional nutritional supplements to the Arabidopsis plants that could induce nitrogen deficiency could also play a role in our study. Nitrogen deficiency down-regulates its metabolism and limits amino acid accumulation in Arabidopsis (North *et al.*, 2009; Ľuptáková *et al.*, 2023). The lower Gln levels in stressed plants, usually reduced under low nitrogen (Fritz *et al.*, 2006), also suggested a combined drought and nitrogen limitation stress. This is further supported by the high Gln:Glu ratio (22, typically ~10), an indicator of nitrogen limitation (Annunziata *et al.*, 2017), due to a more substantial reduction of Glu. This amino acid is the precursor for many secondary metabolites related to plant stress response, such as polyamines, which tend to be accumulated under stress conditions (De Diego *et al.*, 2013; Ľuptáková *et al.*, 2023), as also shown in our study. Furthermore, a recent study performed in barley under combined nitrogen deficiency and drought demonstrated that glutamine synthase (GS; EC 6.3.1.2), which converts Glu to Gln, was up-regulated, together with many other proteins related to biogenic amine metabolism (Li and Wang, 2023).

Regarding acetylated compounds, while well-watered plants accumulated N5AcOrn and N6AcLys, water-stressed plants had higher levels of N2AcOrn and N2AcLys (Fig. 9C). The role of these acetylated amino acids in plant stress responses is not well understood. However, the accumulation of N2AcLys was identified as a marker of drought stress sensitivity in *Elymus sibiricus* (Yu *et al.*, 2022). Levels of N5AcOrn and N6AcLys in *Physcomitrium patens* were found to be sensitive to various abiotic stress conditions and some phytohormones (Bělíček *et al.*, 2023). However, the two isoforms are usually analyzed together (Adio *et al.*, 2011; Hernándiz *et al.*, 2022a).

### Conclusions

A new LC-MS/MS method for analyzing 74 nitrogen-related plant metabolites in two independent chromatographic runs using both HILIC and RP separation has been developed. This method permits fast and effective extraction and chromatographic separation of these analytes from extremely complex plant matrices. Only 2–5 mg of lyophilized plant tissue is needed to obtain data on levels of free amino acids and biogenic amines, as well as their acetylated and methylated derivatives and the levels of aliphatic conjugated and bound biogenic amines. We tested several extraction solvents and purification methods, including SPE and QuEChERS. Surprisingly, no purification was needed to analyze free amino acids, acetylated/methylated amino acids, and acetylated biogenic amines. To distinguish between free, bound, and conjugated biogenic amines, we developed a fractionation procedure that allows us to quantify these different forms separately. In addition, we also developed a new synthetic route for deuterium-labeled biogenic amines. The new method offers high extraction efficiencies and good chromatographic resolution, as well as satisfactory sensitivity and selectivity. Moreover, it has been rigorously validated by measuring metabolite levels in seven plant species at different developmental stages under diverse growth conditions. As such, it has the potential to expedite research on these uncommon metabolites and thereby improve our understanding of their biological roles across different plant species.

## Supplementary data

The following supplementary data are available at *JXB* online.

Table S1. Retention times and optimal MRM transitions of analytes and internal standards separated on the BEH AMIDE column.

Table S2. Retention times and optimal MRM transitions of derivatized biogenic amines and internal standards separated on the BEH C18 column.

Dataset S1. Analyte log ratios used to generate the heatmap presented in Fig. 5.

Dataset S2. Levels of N-related metabolites in seven plant species, Arabidopsis, radish, maize, barley, wheat, tomato, and tobacco.

Dataset S3. Levels of N-related metabolites in tomato seedlings at two developmental stages (cotyledons and true leaves) and Arabidopsis grown under well-watered and drought conditions.

Dataset S4. Absolute intensities of the analytes separated on HILIC column.

Appendix S1. Chemicals and reagents used in the study.

erae129_suppl_Supplementary_Appendixs_S1

erae129_suppl_Supplementary_Tables_S1-2

erae129_suppl_Supplementary_Datasets_S1-S3

## Data Availability

The data supporting this article can be obtained by contacting the corresponding author.
