# Supplementary material for: Comprehensive LC-MS/MS analysis of nitrogen-related plant metabolites
Source: J Exp Bot. 2024 Apr 25;75(17):5390–411. doi: 10.1093/jxb/erae129 (PMC11389842; doi:10.1093/jxb/erae129)
Supplement: erae129_suppl_Supplementary_Appendixs_S1 [file erae129_suppl_supplementary_appendixs_s1.pdf]

Appendix S1.

Chemicals

Standard compounds of the highest available purity were purchased from various chemical companies. 2-Aminoadipic acid (AAA), *N*-acetylhistamine (AcHist), *N*2-acetylornithine (N2AcOrn), *N*-acetylputrescine (AcPut), *N*1-acetylspermine (N1AcSpm), *N*2-acetyllysine (N2AcLys), agmatine (Agm), alanine (Ala), allantoin (All), arginine (Arg), asparagine (Asn), aspartic acid (Asp), β-aminobutyric acid (BABA), β-alanine (BAla), glycine betaine (GB), cadaverine (Cad), cystine (Cis), citrulline (Cit), cysteine (Cys), *N*1,*N*12-diacetylspermine (dAcSpm), diaminopropane (Dap), γ-aminobutyric acid (GABA), glutamine (Gln), glutamic acid (Glu), glycine (Gly), homocysteine (hCys), histidine (His), histamine (Hist), isoleucine (Ile), leucine (Leu), lysine (Lys), methionine (Met), norspermidine (nSpd), ornithine (Orn), phenylalanine (Phe), putrescine (Put), *S*-(5′-adenosyl)-l-methionine (SAM), serine (Ser), spermidine (Spd), spermine (Spm), threonine (Thr), tryptophan (Trp), tryptamine (Tryp), tyrosine (Tyr), tyramine (Tyra), valine (Val), and *trans*-2-hydroxyproline (Hyp) were purchased from Merck (Czech Republic). *N*-Acetyl-β-alanine (AcBAla), *N*-acetylglutamic acid (AcGlu), and proline (Pro) were purchased from Alfa Aesar (USA). *N*-Acetyltyramine (AcTyra) was bought from Apollo Scientific (UK) and thermospermine (tSpm) from Astatech Inc (USA). Allantoic acid or allantoate (Alla) and *N*5-Acetylornithine (N5AcOrn) were purchased from Biosynth Carbosynth (UK), *N*2,*N*6-diacetyllysine (dAcLys) were obtained from BLDPharm (Germany) and *N*2,*N*5-diacetylornithine (dAcOrn) from BOC Sciences (USA). *N*4-Acetylagmatine (AcAgm), *N*-acetamidobutiric acid (AcGABA), *N*1-acetylspermidine (N1AcSpd), *S*-adenosylhomocysteine (SAH), and *L*-homoarginine (hArg) were bought from Cayman Chemical Company (USA), while *N*,*N*-diacetyldiaminopropane (dAcDap) was purchased from CF Plus Chemicals (Czech Republic). *N*-acetylnorspermidine (AcnSpd) and β-alanine betaine (BAB) were obtained from ChemSpace (Latvia), and *N*8-acetylspermidine (N8AcSpd) and *homo*-spermidine (hSpd) from ChemCruz (USA). L-Pyroglutamic acid (pGlu) and stachydrine (Sta) were purchased from Gentham Life Science (UK), and *N*6-acetyllysine (N6AcLys) from MP Biomedicals (USA). Finally, *N*-acetyltryptamine (AcTryp), *N*-acetylcadaverine (AcCad), *N*2-acetylcitrulline (AcCit), and *N*-Phenethylacetamide (AcPEA) were purchased from Toronto Research Chemicals (Canada).

Deuterated γ-aminobutyric acid-2,2,3,3,4,4-d_6 (d_6-GABA), leucine-2,3,3,4,5,5,5,5′,5′,5′-d_{10} (d_{10}-Leu), glutamic acid-2,3,3,4,4-d_5 (d_5-Gln), glutamine-2,3,3,4,4-d_5 (d_5-Glu), proline-$^{13}C_5$,^{15}N ($^{13}C_5$,^{15}N-Pro), and threonine-$^{13}C_4$,^{15}N,2,3,4,4,4-d_5 ($^{13}C_4$,^{15}N,2d_5-Thr) were bought from Merck (Czech Republic), while alanine-2,3,3,3-d_4 (Ala IS) was obtained from Cambridge Isotope Laboratories, Inc. (UK).

Other labeled standards, namely *N*-acetyldiaminopropane-1,1-d_2 (d_2-AcDap), *N*,*N*-diacetyldiaminopropane-1,1,2,2,3,3-d_6 (d_6-dAcDap), diaminopropane-1,1,2,2,3,3-d_6 (d_6-Dap), *N*1-acetylspermidine-2,2,3,3,4,4-d_6 (d_6-N1AcSpd), and *N*1-acetylspermine-2,2,3,3,4,4-d_6 (d_6-N1AcSpm) were synthesized in our laboratories, as was AcDap. Protocols for the synthesis of these compounds are presented below.

The acetonitrile (ACN) and methanol (MeOH) used in the mobile phases were of LCMS grade and purchased from Merck (Germany). Formic acid (FA), hydrochloric acid (HCl) solution (36%), ammonium formate (AF), ammonium hydroxide (NH_4OH) solution (25%), sodium hydroxide (NaOH), sodium chloride (NaCl), benzoyl chloride, trichloroacetic acid (TCA), 96% ethanol (EtOH), and diethyl ether were of the highest purity available and purchased from Sigma-Aldrich (Czech Republic).
