# Supplementary material for: Comprehensive LC-MS/MS analysis of nitrogen-related plant metabolites
Source: J Exp Bot. 2024 Apr 25;75(17):5390–411. doi: 10.1093/jxb/erae129 (PMC11389842; doi:10.1093/jxb/erae129)
Supplement: erae129_suppl_Supplementary_Tables_S1-2 [file erae129_suppl_supplementary_tables_s1-2.pdf]

Supplementary Table S1. Retention times and optimal MRM conditions of analytes and internal standards separated on the BEH AMIDE column.

#	Compound	Rt (min) Mean±SD	MRM transitions (collision energy V)	Rel. intensity (%)
1.	AcPEA	1.357±0.051	164.15>104.95 (-15)	100
			164.15>77.00 (-35)	35
			164.15>79.00 (-25)	19
2.	AcTryp	1.395±0.074	203.05>144.00 (-15)	100
			203.05>117.10 (-30)	23
			203.05>115.10 (-36)	23
3.	AcTyra	1.669±0.059	180.00>121.00 (-15)	100
			180.00>77.00 (-36)	60
			180.00>103.10 (-25)	58
4.	AcGABA	2.3070.071	146.00>86.00 (-10)	100
			146.00>43.00 (-27)	5
			146.00>69.00 (-25)	5
5.	AcBAla	2.416±0.071	131.95>114.20 (-13)	100
			131.95>72.00 (-14)	95
			131.95>90.00 (-12)	35
6.	d_6-dAcDap	2.917±0.030	165.17>106.00 (-15)	100
			165.17>74.00 (-22)	14
			165.17>32.05 (-29)	6
7.	dAcDap	2.968±0.037	159.00>100.00 (-15)	100
			159.00>72.00 (-22)	36
			159.00>43.00 (-41)	20
8.	PGlu	3.718±0.054	130.15>84.05 (-15)	100
			130.15>56.10 (-25)	17
			130.15>41.20 (-23)	10
9.	dAcLys	4.355±0.075	231.15>84.00 (-30)	100
			231.15>126.00 (-15)	64
			231.15>213.10 (-9)	8
10.	AcGlu	4.664±0.067	190.00>84.00 (-25)	100
			190.00>130.20 (-13)	45
			190.00>102.20 (-16)	7
11.	dAcOrn	5.022±0.138	217.05>70.00 (-25)	100
			217.05>112.10 (-15)	70
			217.05>199.20 (-10)	13
12.	Tryp	6.647±0.081	160.95>144.10 (-14)	100
			160.95>115.10 (-34)	46
			160.95>116.95 (-24)	41
13.	All	6.885±0.128	156.90>97.10 (14)	100
			156.90>42.05 (10)	90
			156.90>114.10 (14)	35
14.	AcCit	6.969±0.113	218.05>70.00 (-30)	100
			218.05>201.05 (-10)	86
			218.05>159.10 (-15)	50
15.	Tyra	7.301±0.101	137.90>121.05 (-14)	100
			137.90>77.00 (-28)	85
			137.90>91.05 (-23)	36
16.	AcAgm	8.416±0.066	173.00>114.00 (-15)	100
			173.00>72.00 (-20)	96
			173.00>156.10 (-15)	31
17.	AcHist	8.451±0.073	154.00>94.95 (-20)	100

			154.00>68.00 (-30)	15
			154.00>112.20 (-16)	9
18.	AcCad	8.626±0.069	145.00>86.00 (-15)	100
			145.00>128.15 (-16)	20
			145.00>72.00 (-14)	17
19.	BAB	8.794±0.075	132.00>58.10 (-30)	100
			132.00>59.15 (-22)	17
			132.00.60.25 (-18)	9
20.	d_2-AcDap	8.826±0.075	119.31>102.00 (-12)	100
			119.31>74.00 (-15)	20
			119.31>42.95 (-29)	15
21.	AcDap	8.830±0.083	117.05>100.05 (-10)	100
			117.05>71.95 (-16)	24
			117.05>30.00 (-20)	16
22.	d_{10}-Leu	8.945±0.056	141.95>96.10 (-12)	100
23.	Leu	8.967±0.067	132.10>86.05 (-12)	100
			132.10>44.15 (-23)	20
24.	Phe	8.989±0.058	165.90>120.10 (-15)	100
			65.90>103.10 (-27)	58
			165.90>77.00 (-40)	56
25.	AcPut	9.049±0.060	131.00>72.00 (-16)	100
			131.00>114.20 (-15)	61
			131.00>30.05 (-23)	14
26.	Trp	9.081±0.053	205.10>188.20 (-11)	100
			205.10>118.00 (-25)	81
			205.10>146.00 (-18)	71
27.	d_6-GABA	9.125±0.056	110.15>93.00 (-14)	100
			110.15>49.05 (-22)	69
			110.15>46.00 (-23)	26
28.	GABA	9.137±0.062	104.10>87.10 (-14)	100
			104.10>45.05 (-23)	60
			104.10>69.00 (-16)	37
29.	Ile	9.140±0.091	132.10>86.15 (-12)	100
			132.10>68.95 (-16)	20
30.	Sta	9.157±0.063	144.15>58.20 (-24)	100
			144.15>84.10 (-22)	70
			144.15>42.00 (-49)	20
31.	GB	9.173±0.084	117.95>58.10 (-25)	100
			117.95>59.20 (-18)	33
			117.95>42.10 (-53)	8
32.	BABA	9.314±0.098	104.10>45.05 (-24)	100
			104.10>41.00 (-30)	57
33.	Met	9.604±0.048	149.90>56.10 (-17)	100
			149.90>61.00 (-23)	85
			149.90>104.00 (-13)	56
34.	Val	9.856±0.048	118.00>72.00 (-10)	100
			118.00>55.05 (-20)	42
35.	hCys	9.873±0.148	136.10>90.05 (-11)	100
			136.10>56.20 (-18)	58
			136.10>47.05 (-29)	17
36.	Pro	9.981±0.043	116.10>70.05 (-7)	100
			116.10>43.05 (-22)	4
37.	$^{13}C_5,^{15}N$-Pro	9.983±0.083	122.10>75.00 (-16)	100
			122.10>46.10 (-30)	3

			122.10>30.10 (-38)	3
38.	BAla	10.116±0.044	90.10>72.10 (-12)	100
			90.10>30.10 (-13)	97
			90.10>45.15 (-33)	33
39.	Cys	10.171±0.141	121.90>38.85 (-14)	100
			121.90>81.00 (-7)	42
40.	Tys	10.180±0.040	182.10>91.10 (-28)	100
			182.10>136.00 (-15)	48
			181.10>165.15 (-13)	35
41.	Alla	10.283±0.091	176.95>61.10 (-11)	100
			176.95>134.10 (-11)	47
			176.95>74.10 (-18)	16
42.	N2AcLys	10.545±0.050	189.05>84.00 (-25)	100
			189.05>126.00 (-15)	13
			189.05>56.05 (-41)	5
43.	d_4-Ala	11.067±0.054	94.15>48.10 (-13)	100
			94.15>45.10 (-40)	6
			94.15>30.10 (-370	3
44.	Ala	11.071±0.036	90.10>44.10 (-12)	100
			90.10>45.00 (-31)	8
45.	N2AcOrn	11.132±0.056	175.00>70.00 (-25)	100
			175.00>112.10 (-10)	10
			175.00>43.00 (-40)	5
46.	Hpr	11.296±0.031	132.90>87.10 (-15)	100
			132.90>69.00 (-22)	86
			132.90>68.05 (-22)	23
47.	Aaa	11.359±0.024	161.90>98.00 (-15)	100
			161.90>55.10 (-26)	53
			161.90>121.00 (-10)	9
48.	N6AcLys	11.426±0.022	189.05>84.00 (-25)	100
			189.05>129.00 (-15)	21
			189.05>56.05 (-40)	1
49.	$^{13}C_4$,^{15}N,d_5-Thr	11.582±0.072	129.90>80.00 (-13)	100
			129.90>65.10 (-18)	24
50.	Thr	11.585±0.066	120.10>74.00 (-14)	100
			120.10>102.10 (-15)	22
51.	N5AcOrn	11.645±0.027	175.00>70.00 (-25)	100
			175.00>115.20 (-12)	2
			175.00>116.20 (-16)	1
52.	Gly	11.698±0.134	76.10>30.00 (-11)	100
			76.10>48.00 (-14)	3
53.	d_5-Glu	11.749±0.065	152.95>88.00 (-18)	100
			152.95>89.20 (-16)	60
			152.95>135.10 (-14)	28
54.	Glu	11.759±0.029	147.90>84.10 (-17)	100
			147.90>56.00 (-20)	17
55.	d_5-Gln	12.273±0.022	151.90>88.00 (-18)	100
			151.90>135.10 (-14)	88
			151.90>89.20 (-19)	72
56.	Gln	12.262±0.033	146.90>84.10 (-18)	100
			146.90>130.10 (-15)	37
			146.90>56.05 (-29)	25
57.	Ser	12.348±0.043	106.00>60.00 (-15)	100
			106.10>88.00 (-11)	21

58.	Asp	12.454±0.101	134.00>88.00 (-10)	100
			134.00>74.00 (-12)	66
59.	Asn	12.523±0.021	132.90>74.00 (-16)	100
			132.90>87.15 (-11)	58
			132.90>88.10 (-12)	12
60.	Cad	12.551±0.024	103.10>86.10 (-12)	100
			103.10>69.00 (-17)	13
			103.10>41.05 (-23)	10
61.	Cit	12.707±0.015	175.90>70.05 (-23)	100
			175.90>159.20 (-14)	50
			175.90>113.05 (-17)	29
62.	SAH	12.930±0.035	385.00>136.15 (-21)	100
			385.00>134.15 (-19)	75
			385.00>88.00 (-45)	68
63.	dAcSpm	12.934±0.048	287.20>100.00 (-25)	100
			287.20>171.05 (-15)	70
			287.20>72.05 (-35)	14
64.	d_6-N1AcSpd	13.281±0.067	195.10>72.15 (-22)	100
			195.10>107.20 (-17)	85
			195.10>106.15 (-16)	78
65.	N1AcSpd	13.285±0.071	188.00>72.05 (-17)	100
			188.00>100.10 (-16)	44
66.	Hist	13.453±0.059	111.95>95.05 (-15)	100
			111.95>68.00 (-22)	30
			111.95>41.00 (-27)	15
67.	Agm	13.551±0.026	131.00>72.00 (-16)	100
			131.00>60.00 (-12)	39
			131.00>114.10 (-15)	19
68.	AcNSsd	13.554±0.070	174.25>100.10 (-16)	100
			174.25>72.00 (-23)	21
			174.25>58.25 9-22)	10
69.	Put	13.800±0.028	89.15>72.00 (-12)	100
			89.15>30.10 (-24)	11
			89.15>55.05 (-22)	2
70.	hArg	13.636±0.040	189.15>84.05 (-22)	100
			189.15>144.25 (-16)	43
			189.15>85.10 (-24)	11
71.	Arg	13.865±0.036	175.00>70.05 (-23)	100
			175.00>116.10 (-15)	67
72.	N8AcSpd	13.719±0.094	188.80>86.15 (-22)	100
			188.80>87.00 (-22)	3
73.	His	13.995±0.028	155.90>110.15 (-16)	100
			155.90>83.10 (-24)	45
74.	Lys	14.055±0.041	146.90>84.10 (-18)	100
			146.90>130.00 (-15)	20
			146.90>56.15 (-28)	18
75.	d_6-Dap	14.116±0.029	81.15>64.05 (-13)	100
			81.15>31.10 (-23)	20
			81.15>46.00 (-22)	14
76.	Dap	14.119±0.092	75.25>58.10 (-13)	100
			72.25>30.00 (-23)	21
			75.25>41.00 (-18)	15
77.	Orn	14.161±0.040	133.10>70.00 (-22)	100
			133.10>116.00 (-15)	84

			133.10>43.15 (-33)	59
78.	SAM	14.462±0.038	399.45>249.95 (-15)	100
			399.45>136.10 (-25)	65
			399.45>97.20 (-30)	60
79.	Cis	14.743±0.025	241.00>74.00 (-29)	100
			214.00>151.95 (-14)	62
80.	hSpd	15.689±0.367	160.00>72.00 (-16)	100
			160.00>126.15 (-16)	18
			160.00>30.05 (-35)	6
81.	Spd	15.725±0.311	145.95>72.00 (-16)	100
			145.95>84.10 (-23)	27
			145.95>112.05 (-16)	20
82.	nSpd	15.824±0.309	131.95>98.00 (-17)	100
			131.95>58.10 (-17)	70
			131.95>70 (-24)	44
83.	d_6-N1AcSpm	15.824±0.317	252.10>106.20 (-22)	100
			252.10>113.20 (-21)	80
			252.10>178.25 (-14)	70
84.	N1AcSpm	15.903±0.352	245.10>100.10 (-22)	100
			245.10>112.15 (-21)	75
			245.10>84.20 (-36)	35
85.	Spm	15.426±0.362	203.05>112.05 (-21)	100
			203.05>84.05 (-30)	82
			203.05>129.15 (-14)	50

Supplementary Table S2. Retention times and optimal MRM conditions of derivatized biogenic amines and internal standards separated on the BEH C18 column.

#	Compound	Rt (min)<br>Mean±SD	MRM transitions<br>(collision energy V)	Rel. intensity<br>(%)
1.	Put	1.638±0.012	297.10>105.10 (25)	100
			297.10>77.05 (50)	75
			297.10>176.00 (16)	53
2.	d_6-Dap	1.679±0.022	289.10>105.00 (26)	100
			298.10>77.00 (50)	73
			289.10>168.10 (16)	59
3.	Dap	1.686±0.023	282.95>104.95 (25)	100
			282.95>77.00 (55)	78
			282.95>162.05 (15)	67
4.	Cad	1.739±0.012	311.10>104.95 (24)	100
			311.10>77.05 (52)	71
			311.10>190.20 (15)	32
5.	nSpd	2.080±0.033	444.15>105.05 (46)	100
			444.15>162.00 (23)	87
			444.15>322.00 (29)	77
6.	Spd	2.231±0.033	458.20>105.10 (44)	100
			458.20>162.16 (29)	65
			458.20>336.30 (19)	64
7.	hSpd	2.429±0.024	472.20>274.20 (22)	100
			472.20>105.05 (43)	78
			472.20>176.05 (29)	40
8.	tSpm	3.121±0.017	619.25>105.00 (54)	100
			619.25>162.00 (34)	65
			619.25>337.20 (25)	31
9.	Spm	3.315±0.027	619.25>497.30 (26)	100
			619.25>162.00 (40)	81
			619.25>105.00 (55)	9
10.	Agm	4.404±0.034	443.00>104.85 (33)	100
			443.00>375.00 (13)	15
